# Survival prediction models since liver transplantation - comparisons between Cox models and machine learning techniques

**DOI:** 10.1186/s12874-020-01153-1

**Published:** 2020-11-16

**Authors:** Georgios Kantidakis, Hein Putter, Carlo Lancia, Jacob de Boer, Andries E. Braat, Marta Fiocco

**Affiliations:** 1 Mathematical Institute (MI) Leiden University, Niels Bohrweg 1, Leiden, 2333 CA the Netherlands; 2 Department of Biomedical Data Sciences, Section Medical Statistics, Leiden University Medical Center (LUMC), Albinusdreef 2, Leiden, 2333 ZA The Netherlands; 3 Department of Statistics, European Organisation for Research and Treatment of Cancer (EORTC) Headquarters, Ave E. Mounier 83/11, Brussels, 1200 Belgium; 4 Department of Surgery, Leiden University Medical Center (LUMC), Albinusdreef 2, Leiden, 2333 ZA the Netherlands; 5 Trial and Data Center, Princess Máxima Center for pediatric oncology (PMC), Heidelberglaan 25, Utrecht, 3584 CS the Netherlands

**Keywords:** Random survival forest, Neural networks, Predictive performance, Risk factors, Post-transplantation, Survival analysis

## Abstract

**Background:**

Predicting survival of recipients after liver transplantation is regarded as one of the most important challenges in contemporary medicine. Hence, improving on current prediction models is of great interest.Nowadays, there is a strong discussion in the medical field about machine learning (ML) and whether it has greater potential than traditional regression models when dealing with complex data. Criticism to ML is related to unsuitable performance measures and lack of interpretability which is important for clinicians.

**Methods:**

In this paper, ML techniques such as random forests and neural networks are applied to large data of 62294 patients from the United States with 97 predictors selected on clinical/statistical grounds, over more than 600, to predict survival from transplantation. Of particular interest is also the identification of potential risk factors. A comparison is performed between 3 different Cox models (with all variables, backward selection and LASSO) and 3 machine learning techniques: a random survival forest and 2 partial logistic artificial neural networks (PLANNs). For PLANNs, novel extensions to their original specification are tested. Emphasis is given on the advantages and pitfalls of each method and on the interpretability of the ML techniques.

**Results:**

Well-established predictive measures are employed from the survival field (C-index, Brier score and Integrated Brier Score) and the strongest prognostic factors are identified for each model. Clinical endpoint is overall graft-survival defined as the time between transplantation and the date of graft-failure or death. The random survival forest shows slightly better predictive performance than Cox models based on the C-index. Neural networks show better performance than both Cox models and random survival forest based on the Integrated Brier Score at 10 years.

**Conclusion:**

In this work, it is shown that machine learning techniques can be a useful tool for both prediction and interpretation in the survival context. From the ML techniques examined here, PLANN with 1 hidden layer predicts survival probabilities the most accurately, being as calibrated as the Cox model with all variables.

**Trial registration:**

Retrospective data were provided by the Scientific Registry of Transplant Recipients under Data Use Agreement number 9477 for analysis of risk factors after liver transplantation.

**Supplementary Information:**

The online version contains supplementary material available at (doi:10.1186/s12874-020-01153-1).

## Background

Liver transplantation (LT) is the second most common type of transplant surgery in the United States after kidney [1]. Over the last decades, the success of liver transplants has improved survival outcome for a large number of patients suffering from chronic liver disease everywhere on earth [2]. Availability of donor organs is a major limitation especially when compared with the growing demand of liver candidates due to the enlargement of age limits. Therefore, improvement on current prediction models for survival since LT is important.

There is an open discussion about the value of machine learning (ML) versus statistical models (SM) within clinical and healthcare practice [3–7]. For survival data, the most commonly applied statistical model is the Cox proportional hazards regression model [8]. This model allows a straightforward interpretation, but is at the same time restricted to the proportional hazards assumption. On the other hand, ML techniques are assumption-free and data adaptive which means that they can be effectively employed for modelling complex data. In this article, the results between SM and ML techniques are assessed based on a 3-stage comparison: predictive performance for large sample size/large number of covariates, calibration (absolute accuracy) which is often neglected, and interpretability in terms of the most prognostic factors identified. Advantages and disadvantages for each method are detailed.

ML techniques need a precise set of operating conditions to perform well. It is important that a) the data have been adequately processed so that the inputs allow for good learning, b) modern method is applied using state-of-the-art programming software and c) proper tuning of the parameters is performed to avoid sub-optimal or default choices for parameters which downgrade the algorithm’s performance. Danger of overfitting is associated with ML approaches (as they employ complex algorithms). A note of caution is required during model training to prevent from overfitting, e.g. the selection of suitable hyper-parameters. Needless to say, overfitting might also occur with a traditional model if it is too complex (estimation of too many parameters) thus limiting generalizability outside training instances.

Neural networks have been commonly applied in healthcare. Consequently, different approaches for time-to-event endpoints are present in the literature. Biganzoli et al. proposed a partial logistic regression approach of feed forward neural networks (PLANN) for flexible modelling of survival data [9]. By using the time interval as an input in a longitudinally transformed feed forward network with logistic activation and entropy error function, they estimated smoothed discrete hazards at each time interval in the output layer. This is a well known approach for modelling survival neural networks [10]. In 2000, Xiang et al. [11] compared the performance of 3 existing neural network methods for right censored data (the Faraggi-Simon [12], the Liestol-Andersen-Andersen [13] and a modification of the Buckley-James method [14]) with Cox models in a Monte Carlo simulation study. None of the networks outperformed the Cox models and they only performed as good as Cox for some scenarios. Lisboa et al. extended the PLANN approach introducing a Bayesian framework which can perform Automatic Relevance Determination for survival data (PLANN-ARD) [15]. Several applications of the PLANN and the PLANN-ARD methods can be found in the literature [16–19]. They show potential for neural networks in systems with non-linearity and complex interactions between factors. Here extensions of the PLANN approach for big LT data are examined.

The clinical endpoint of interest for this study is overall graft-survival defined as the time between LT and graft-failure or death. Predicting survival after LT is hard as it depends on many factors and is associated with donor, transplant and recipient characteristics whose importance changes over time and per outcome measure [20]. Models that combine donor and recipient characteristics have usually better performance for predicting overall graft-survival and particularly those that include sufficient donor risk factors have better performance for long-term graft survival [21]. The aims of this manuscript can be summarised as: i) potential role of ML as a competitor of traditional methods when complexity of the data is high (large sample size, high dimensional setting), ii) identification of potential risk factors using 2 ML methods (random survival forest, survival neural networks) complementary to the Cox model, iii) use of variable selection methods to compare their predictive ability with the models including the non-reduced set of variables, iv) evaluation of predictions and goodness of fit, and v) clinical relevance of the findings (potential for medical applications).

The paper is organized as follows. “Methods” section presents details about data collection and the imputation technique, SMs and ML. Further sections discuss model training, predictive performance assessment on test data, and details about interpretability of the models. Comparisons between models based on global performance measures, prediction error curves, variable importance and calibration plots are discussed in the “Results” section. The article is concluded by the “Discussion” section about findings, limitations of this work and future perspectives. All analyses were performed in R programming language version 3.5.3 [22]. Preliminary results were presented at 40th Annual Conference of the International Society for Clinical Biostatistics [23].

## Methods

An analysis is presented on survival data after LT based on 62294 patients from the United States. Information was collected from the United Network of Organ Sharing (UNOS)^1^. After extensive pre-processing from a set of more than 600 covariates, 97 variables were included in the final dataset based on clinical and statistical considerations (see Additional file 1); 52 donor and 45 liver recipient characteristics (missing values were imputed). As the UNOS data is large in both number of observations and covariates, it is of interest to see how ML algorithms - which are able to capture naturally multi-way interactions between variables and can deal with big datasets - will perform compared to Cox models. The clinical endpoint is overall graft-survival (OGS) the time between LT and graft-failure or death. The choice for this endpoint was made for two reasons 1) it is of primary interest for clinicians and 2) it is the most appropriate outcome measure to evaluate the efficacy of LT, because it incorporates both patient mortality and survival of the graft [21].

This section is divided into different subsections including the necessary components of analyses for OGS (provided in “Results” section). We discuss in detail both Cox models and ML techniques (Random Survival Forest, Survival Neural Networks). Elements of how the models were trained and how the predictive performance was assessed on the test data are presented. More technical details are provided in the supplementary material. We conclude this extensive section with a focus on methods to extract interpretation for the ML approaches.

### Data collection and imputation technique

UNOS manages the Organ Procurement and Transplantation Network (OPTN) and together they collect, organise and maintain data of statistical information regarding organ transplants in the Scientific Registry of Transplant Recipients (SRTR) database^2^. SRTR gathers data from local Organ Procurement Organisations (OPO) and from OPTN (primary source). It includes data from transplantations performed in the United States from 1988 onwards. This information is used to set priorities and seek improvements in the organ donation process.

The data provided by UNOS included 62294 patients who underwent LT surgery from 2005 to 2015 (project under DUA number 9477). Standard analysis files contained 657 variables for both donors and patients (candidates and recipients). Among these, 97 candidate risk factors - 52 donor and 45 patient characteristics - were pre-selected before carrying out analysis. This resulted in a final dataset with 76 categorical and 21 continuous variables amounting to 2.2% missing data overall. The percentage of missing values for each covariate varied from 0 to 26.61% (no missing values for 26 covariates, up to 1% missingness for 51 covariates, 1 to 10% for 11 variables, 10 to 25% for 7 variables and 25 to 26.61% for only 2 variables). Analysis on the complete case would reduce the available sample size from 62294 to 33394 patients leading to a huge waste of data. Furthermore, this could lead to invalid results (underestimation or overestimation of survival) if the excluded group of patients represents a subgroup from the entire sample [24]. To reconstruct the missing values the missForest algorithm [25] was applied for both continuous and categorical variables. This is a non-parametric imputation method that does not make explicit assumptions about the functional form of the data and builds a random forest model for each variable (500 trees were used). It specifies the model to predict missing values by using information based on the observed values. It is the most exhaustive and accurate of all random forests algorithms used for missing data imputation, because all possible variable combinations are checked as responses.

### Cox proportional Hazard regression models

In survival analysis, the focus is on the time till the occurrence of the event of interest (here graft-failure or death). The Cox proportional hazards model is usually employed to estimate the effect of risk factors on the outcome of interest [8].

Data with sample size *n* consist of the independent observations from the triple (*T*,*D*,*X*) i.e. (*t*_1_,*d*_1_,*x*_1_),⋯,(*t*_*n*_,*d*_*n*_,*x*_*n*_). For the *i*^*th*^ individual, *t*_*i*_ is the survival time, *d*_*i*_ the indicator (*d*_*i*_=1 if the event occurred and *d*_*i*_=0 if the observation is right censored) and *x*_*i*_ is the vector of predictors (*x*_1_,⋯,*x*_*p*_). The hazard function of the Cox model with time-fixed covariates is as follows:

1 $$\begin{array}{@{}rcl@{}} h(t|X) = h_{0}(t)\exp\left(X^{T}\boldsymbol{\beta}\right), \end{array} $$

where *h*(*t*|*X*) is the hazard at time t given predictor values X, *h*_0_(*t*) is an arbitrary baseline hazard and ***β***=(*β*_1_,⋯,*β*_*p*_) is a parameter vector.

The corresponding partial likelihood can be written as:

2 $$\begin{array}{@{}rcl@{}}  L(\boldsymbol{\beta}) = \prod_{i=1}^{D} \frac{\exp{\left(\sum_{k = 1}^{p} \beta_{k} X_{ik}\right)}}{\sum_{j\in R(t_{i})} \exp{\left(\sum_{k = 1}^{p} \beta_{k} Z_{jk}\right)}}, \end{array} $$

where *D* is the set of failures, and *R*(*t*_*i*_) is the risk set at time *t*_*i*_ of all individuals who are still in the study at the time just before time *t*_*i*_. This function is then maximised over ***β*** to estimate the model parameters.

Two other Cox models were employed 1) a Cox model with a backward elimination and 2) a penalised Cox regression with the Least Angle and Selection Operator (LASSO). Both models have been widely used for variable selection. We aim to compare these more parsimonious models versus a Cox model with all variables in terms of predictive performance. For the first, a numerically stable version of the backward elimination on factors was applied using a method based on Lawless and Singhal (1978) [26]. This method estimates the full model and computes approximate Wald statistics by computing conditional maximum likelihood estimates - assuming multivariate normality of estimates. Factors that require multiple degrees of freedom are dropped or retained as a group.

The latter approach uses a combination of selection and regularisation [27]. Denote the log-partial likelihood by *ℓ*(***β***)=*logL*(***β***). The vector ***β*** is estimated via the criterion:

3 $$\begin{array}{@{}rcl@{}}  {\hat{\beta}} =\text{argmin}[\ell({\beta})], \quad \text{subject} \ \text{to} \ \sum_{j = 1}^{p} |\beta_{j}| \leq s \end{array} $$

with *s* a user specified positive parameter.

Equation (3) can also be rewritten as

4 $$\begin{array}{@{}rcl@{}}  {\hat{\beta}} = \text{argmin}{\beta} \left(\ell (\beta) + \lambda_{LASSO} \sum_{j = 1}^{p} |\beta_{j}| \right). \end{array} $$

The quantity $\sum _{j = 1}^{p} |\beta _{j}| $ is also known as the *L*_1_-norm and performs regularisation to the log-partial likelihood. The term *λ*_*LASSO*_ is a non-negative constant that assigns the amount of penalisation. Larger values for the parameter mean larger penalty to the *β*_*j*_ coefficients and enlarged shrinkage towards zero.

The tuning parameter *s* in Eq. (3) or equivalently parameter *λ*_*Lasso*_ in Eq. (4) is the controlling mechanism for the variance of the model. Higher values reduce further the variance but introduce at the same time more bias (variance-bias trade off). To find a suitable value for this parameter 5-fold cross-validation was performed to minimise the prediction error; here in terms of the cross-validated log-partial likelihood (CVPL) [28]

5 $$\begin{array}{@{}rcl@{}}  \textit{CVPL}(s) = \sum_{i = 1}^{n} \left(\ell \left(\hat{\beta}_{(-i)}(s)\right) - \ell_{{(-i)}}\left(\hat{\beta}_{(-i)}(s)\right) \right), \end{array} $$

where *ℓ*_(−*i*)_(*β*) is the partial log-likelihood of Eq. (2) when individual *i* is excluded. Therefore, the term $\ell (\hat {\beta }_{(-i)}) - \ell _{{(-i)}}\left (\hat {\beta }_{(-i)}\right)$ represents the contribution of observation *i*. The value that maximizes *ℓ*_(−*i*)_(*β*_(−*i*)_) is denoted by $\hat {\beta }_{(-i)}$.

### Random forests for survival analysis

Random Survival Forests (RSFs) are an ensemble tree method for survival analysis of right censored data [29] adapted from random forests [30]. The main idea of random forests is to get a series of decision trees - which can capture complex interactions but are notorious for their high variance - and obtain a collection averaging their characteristics. In this way weak learners (the individual trees) are turned into strong learners (the ensemble) [31].

For RSFs, randomness is introduced in two ways: bootstrapping a number of patients at each tree $\mathcal {B}$ times and selecting a subset of variables for growing each node. During growing each survival tree, a recursive application of binary splitting is performed per region (called node) on a specific predictor in such a way that survival difference between daughter nodes is maximised and difference within them is minimised. Splitting is terminated when a certain criterion is reached (these nodes are called terminal). The most commonly used splitting criteria are the log-rank test by Segal [32] and the log-rank score test by Hothorn and Lausen [33]. Each terminal node should have at least a pre-specified number of unique events. Combining information from the $\mathcal {B}$ trees, survival probabilities and ensemble cumulative hazard estimate can be calculated using the Kaplan-Meier and Nelson-Aalen methodology, respectively.

The fundamental principle behind each survival tree is the conservation of events. It is used to define ensemble mortality, a new type of predicted outcome for survival data derived from the ensemble cumulative hazard function (comparable to the prognostic index based on the Cox model). This principle asserts that the sum of estimated cumulative hazard estimate over time is equal to the total number of deaths, therefore the total number of deaths is conserved within each terminal node $\mathcal {H}$ [29]. RSFs can handle both data with large sample size and vast number of predictors. Moreover, they can reach remarkable stability combining the results of many trees. However, combining an ensemble of trees downgrades significantly the intuitive interpretation of a single tree.

### Survival neural networks

Artificial neural networks (ANNs) are a machine learning method able to model non-linear relationships between prognostic factors with great flexibility. These systems are inspired from biological neural networks that aimed at imitating the human brain activity [34]. A ANN has a layered structure and is based on a collection of connected units called nodes or neurons which comprise a layer. The input layer picks up the signals and passes them through transformation functions to the next layer which is called “hidden”. A network may have more than one hidden layer that connects with the previous and transmit signals towards the output layer. Connections between artificial neurons are called edges. Artificial neurons and edges have a weight (connection strength) which adjusts as learning proceeds. It increases or decreases the strength of the signal of each connection according to its sign. For the purpose of training, a target is defined, which is the observed outcome. The simplest form of a NN is the single layer feed-forward perceptron with the input layer, one hidden layer and the output layer [35].

The application of NNs has been extended to survival analysis over the years [13]. Different approaches have been considered; some model the survival probability $\mathcal {S}(t)$ directly or the unconditional probability of death $\mathcal {F}(t)$ whereas other approaches estimate the conditional hazard *h*(*t*) [10]. They can be distinguished according to the method used to deal with the censoring mechanism. Some networks have *k* output nodes [36] - where *k* denotes *k* separate time intervals - while others have a single output node.

In this research, the method of Biganzoli was applied, which specifies a partial logistic feed-forward artificial neural network (PLANN) with a single output node [9]. This method uses as inputs the prognostic factors and the survival times to increase the predictive ability of the model. Data have to be transformed into a longitudinal format with the survival times being divided into a set of *k* non-overlapping intervals (months or years) *I*_*k*_=(*τ*_*k*−1_,*τ*_*k*_], with 0=*τ*_*o*_<*τ*_1_<⋯<*τ*_*k*_ a set of pre-defined time points. In this way, the time component of survival data is taken into consideration. On the training data, each individual is repeated for the number of intervals he/she was observed in the study and on the test data for all time intervals. PLANN provides the discrete conditional probability of dying $\mathcal {P}\left (T \in I_{k} \mid T>\tau _{k-1}\right)$ using as transformation function of both input and output layers the logistic (sigmoid) function:

6 $$\begin{array}{@{}rcl@{}}  f(\eta) &=& \frac{1}{1 + e^{-\eta}}, \end{array} $$

where $\eta = \sum _{i = 1}^{p}w_{i} X_{i}$ is the summed linear combination of the weights *w*_*i*_ of input-hidden layer and the input variables *X*_*i*_ (*i*=1,2,⋯,*p*).

The contribution to the log-likelihood for each individual is calculated all over the intervals one is at risk. The output node is a large target vector with 0 if the event did not occur and 1 if the event occurred in a specific time interval. Therefore, such a network first estimates the hazard for each interval *h*_*k*_=*P*(*τ*_*k*−1_<*T*≤*τ*_*k*_|*T*>*τ*_*k*−1_) and then $S(t) = \prod _{k: t_{k} \leq t} (1 - h_{k})$.

In this work, novel extensions in the specification of the PLANN are tested. Two new transformation functions were investigated for the input-hidden layer the rectified linear unit (ReLU)

7 $$\begin{array}{@{}rcl@{}}  f(\eta) = \eta^{+} = \max{(0, \eta)}, \end{array} $$

which is the most used activation function for NNs and the hyperbolic tangent (tanh)

8 $$\begin{array}{@{}rcl@{}}  f(\eta) &=& \frac{1 - e^{-2\eta}}{1 + e^{-2\eta}}. \end{array} $$

These functions can be seen as different modulators of the degree of non-linearity implied by the input and the hidden layer.

The PLANN was expanded in 2 hidden layers with same node size and identical activation functions for input-hidden 1 and hidden 1 - hidden 2 layers. The *k* non-overlapping intervals of the survival times were treated as *k* separate variables. In this way, the contribution of each interval to the predictions of the model using the relative importance method by Garson [37] and its extension for 2 hidden layers can be obtained (see “Interpretability of the models” section below and Additional file 1).

### Model training

The split sample approach was employed; data was split randomly into two complementary parts, a training set (2/3) and a test set (1/3) under the same event/censoring proportions. To tune a model, 5-fold cross validation was performed in the training set for the machine learning techniques (and for Cox LASSO). Training data was divided into 5 folds. Each time 4 folds were used to train a model and the remaining fold was used to validate its performance and the procedure was repeated for all combination of folds. Tuning of the hyper-parameters was done using grid search and performance of final models was assessed on the test set. Analyses were performed in R programming language version 3.5.3 [22]. Package of implementation for RSFs and NNs as well as technical details regarding the choice of tuning parameters and the cross-validation procedure for each method are provided in Additional File 2.

### Assessing predictive performance on test data

To assess the final predictive performance of the models the concordance index, the Brier score, and the Integrated Brier Score (IBS) were applied.

The most popular measure of model performance in a survival context is the concordance index [38] which computes the proportion of pairs of observations for which the survival times and model predictions order are concordant taking into account censoring. It takes values typically in the range 0.5 - 1 with higher values denoting higher ability of the model to discriminate and 0.5 indicating no discrimination. The C-index cannot be defined for neural network models since it relies on the ordering of individuals according to prognosis and there is no unique ordering between the subjects. At one year individual i may have better survival probability than individual j, but this could be reversed for a different time point.

The C-index provides a rank statistic between the observations that is not time-dependent. Following van Houwelingen and le Cessie [39] a time-dependent prediction error is defined as

9 $$\begin{array}{@{}rcl@{}}  \textit{Brier}\left(y, \hat{S}\left(t_{0}|x\right)\right) &=& \left(y - \hat{S}\left(t_{0}|x\right)\right)^{2}, \end{array} $$

where $\hat {S}(t_{0}|x)$ is the model-based probabilistic prediction for the survival of an individual beyond *t*_0_ given the predictor *x*, and *y*=1{*t*>*t*_0_} is the actual observation ignoring censoring. The expected value with respect to a new observation *Y*_*new*_ under the true model *S*(*t*_0_|*x*) can be written as:

10 $$ {}\begin{aligned} E\left[\textit{Brier}\left(Y_{new}, \hat{S}\left(t_{0}|x\right)\right)\right] &= S\left(t_{0}|x\right) \left(1 - S\left(t_{0}|x\right)\right) \\&\quad+ \left(S\left(t_{0}|x\right) - \hat{S}\left(t_{0}|x\right)\right)^{2}. \end{aligned}  $$

The Brier Score consists of two components: the “true variation” *S*(*t*_0_|*x*)(1−*S*(*t*_0_|*x*)) and the error due to the model $(S(t_{0}|x) - \hat {S}(t_{0}|x))^{2}$. A perfect prediction is only possible if *S*(*t*_0_|*x*)=0 or *S*(*t*_0_|*x*)=1. In practice the two components cannot be separated since the true *S*(*t*_0_|*x*) is unknown.

To assess the performance of a prediction rule in actual data, censored observations before time *t*_0_ must be considered. To calculate Brier Score when censored observations are present, Graf proposed the use of inverse probability of censoring weighting [40]. Then an estimate of the average prediction error of the prediction model $\hat {S}(t|x)$ at time *t*=*t*_0_ is

11 $$  {}\begin{aligned} Err_{Score}\left(\hat{S}, t_{0}\right) &= \frac{1}{n} \sum_{i} 1\left\{d_{i} = 1 \vee t_{i} > t_{0}\right\}\\ &\qquad\frac{Score\left(1\left\{t_{i} > t_{0}\right\}, \hat{S}\left(t_{0}|x_{i}\right)\right)}{\hat{C}\left(\min\left(t_{i}-, t_{0}\right) | x_{i}\right)} \end{aligned}  $$

In (11), $\frac {1}{\hat {C}(\min (t_{i}-, t_{0}) | x_{i})} $ is a weighting scheme known as inverse probability of censoring weighting (IPCW) and *Score* is the Brier Score for the prediction model. It ranges typically from 0 to 0.25 with a lower value meaning smaller prediction error.

Brier score is calculated at different time-points. An overall measure of prediction error is the Integrated Brier Score (IBS) which can be used to summarise the prediction error over the whole range up to the time horizon $\int _{0}^{t_{hor}}Err_{Score}(\hat {S}, t_{0})dt_{0}$ (here *t*_*hor*_ = 10 years) [41]. IBS provides the cumulative prediction error up to *t*_*hor*_ at all available times (*t*^∗^= 1, 2, ⋯, 10 years) and takes values in the same range as the Brier score. In this study, we use IBS as the main criterion to evaluate the predictive ability of all models up to 10 years.

### Interpretability of the models

Interpretation of models is of great importance for the medical community. It is well known that Cox models offer a straightforward interpretation through hazard ratios.

For neural networks with one hidden layer the connection weights algorithm by Garson [37] – later modified by Goh [42] – can provide information about the mechanism of the weights. The idea behind this algorithm is that inputs with larger connection weights produce greater intensities of signal transfer. As a result, these inputs will be more important for the model. Garson’s algorithm can be used to determine relative importance of each input variable, partitioning the weights in the network. Their absolute values are used to specify percentage of importance. Note that the algorithm does not provide the direction of relationships, so it remains uncertain whether the relative importance indicates a positive or a negative effect. For details about the algorithm see [43]. During this work, the algorithm was extended for 2 hidden layers to obtain the relative importance of each variable (for the implementation see algorithm 1 in Additional file 1).

Random survival forest relies on two methods which can provide interpretability: variable importance (VIMP) and minimal depth [44]. The former is associated with the prediction error before and after the permutation of a prognostic factor. Large importance values indicate variables with strong predictive ability. The latter is related to the forest topology as it assesses the predictive value of a variable by computing its depth compared to the root node of a tree. VIMP is more frequently reported than minimal depth in the literature [45]. For both methods interpretation is available only for variable entities and not for each variable level.

## Results

Administrative censoring was applied to the UNOS data at 10 years. Median follow-up is equal to 5.36 years (95% CI: 5.19 - 5.59 years) and it was estimated with reverse Kaplan-Meier [46]. Clinical endpoint is overall graft-survival (OGS). From the total number of patients, 69.1% was alive/censored and 30.9% experienced the event of interest (graft-failure or death). 3 models were used from the Cox family to predict survival outcome: a) a model with all 97 prognostic factors, b) a model with backward selection and c) a model based on the LASSO method for variable selection. Furthermore, 3 machine learning methods were employed: a) a random survival forest, b) a NN with one hidden layer and c) a NN with two hidden layers.

### Comparisons between models

In this section a direct comparison of the 6 models is illustrated in terms of variable importance on the training set and predictive performance on the test set. Specification of the variables with dummy coding included 119 variable levels from the 97 potentially prognostic factors. For NNs - to apply and extend the methodology of Biganzoli - follow-up time was divided into 10 time intervals (0,1],(1,2],⋯, (9,10] denoting years since transplantation. For Cox models and RSF exact time points were used.

Cox model assumes that each covariate has a multiplicative effect in the hazard function (which is constant over time). Estimating a model with 97 prognostic factors leads inevitably to a violation of the proportional hazards assumption for some covariates (17 out of 97 here). This means that hazard ratios for those risk factors are the mean effects on the outcome which is still a valuable information for the clinicians. To consider all possible non-linear effects on interactions leads to a complex model where too many parameters need to be estimated and the interpretability becomes very difficult. On the other hand, ML techniques do not make any assumptions about the data structure and therefore their performance is not affected by the violation of PH. The backward and the LASSO methods selected 28 (out of 97) and 45 predictors (out of 119 dummy coded), respectively. Selection of a smaller set of variables by Cox backward was expected, since it is a greedier (heuristic) method than LASSO penalized regression. The 12 most influential variables for the Cox model with all variables were selected by both methods (see Table 2). 5 of these variables: *re-transplantation*, *donor type*, *log(Total cold ischemic time)*, *diabetes* and *pre-treatment status* violated the PH assumption.

5-fold cross-validation in the training data resulted in the following optimal hyper-parameters combinations for the machine learning techniques:

- For the Random Survival Forest nodesize = 50, mtry = 12, nsplit = 5 and ntree = 300. Stratified bootstrap sub-sampling of half the patients was used per tree (due to the large training time required).
- For the neural network with 1 hidden layer activation function = “sigmoid” (for the input-hidden layer), node size = 85, dropout rate = 0.2, learning rate = 0.2, momentum = 0.9 and weak class weight = 1.
- For the neural network with 2 hidden layers activation function = “sigmoid” (for the input-hidden 1 and the hidden 1-hidden 2 layers), node size = 110, dropout rate = 0.1, learning rate = 0.2, momentum = 0.9 and weak class weight = 1.

#### Global performance measures

The global performance measures on test data are provided in Table 1. Examining the Integrated Brier Score (IBS), the NNs with 1 and with 2 hidden layers have the lowest (IBS = 0.180) followed by the RSF (IBS = 0.182). Cox models have a comparable performance (IBS = 0.183). Therefore, the predictive ability of Cox backward and Cox LASSO is the same as the less parsimonious Cox model with all variables in terms of IBS. The best model in terms of C-index is the Random Survival Forest (0.622) while the Cox models with all variables has slightly worse performance. C-index for Cox backward and Cox LASSO are respectively 0.615 and 0.614.

**Table 1:** Integrated Brier Score (IBS) and C-index on the test data. Neural network 1h and 2h refer to a neural network with one and two hidden layers respectively

	IBS	C-index
Cox all variables	0.183	0.620
Cox backward	0.183	0.615
Cox LASSO	0.183	0.614
RSF	0.182	**0.622**
Neural Network 1h	**0.180**	-
Neural Network 2h	**0.180**	-

Stability of the networks was investigated by rerunning the same models on the test data, and showed that the NN with 1 hidden layer had stable predictive performance and variable importance. In contrast, the NN with 2 hidden layers was quite unstable regarding variable importance. This behavior might be related to the vast amount of weights that had to be trained for this model which can lead to overfitting (in total 26621 connection weights were estimated for a sample size of 41530 patients in long format; whereas for the NN with 1 hidden layer 11136 connection weights). For the RSF, model obtained remarkable stability in terms of performance error after a particular number of trees (ntree = 300 was selected).

### Prediction error curves

**Fig. 1:**
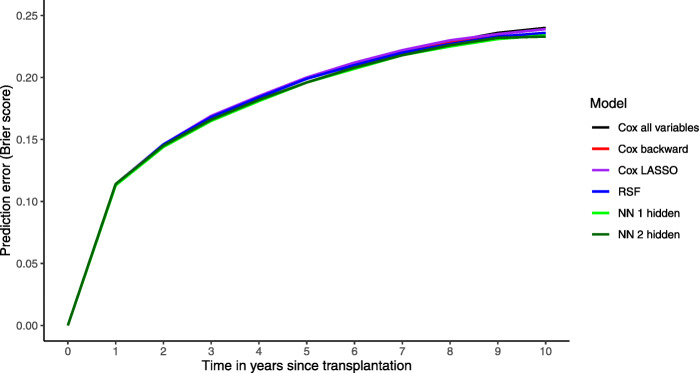
Prediction error curves for all models

Figure 1 shows the average prediction Brier error over time for all models. Small differences can be observed between Cox models and RSF. The NNs with 1 hidden and with 2 hidden layers have almost identical evolution over time achieving better performance than the Cox models and the RSF.

### Variable importance

In this section, the models are compared based on the most prognostic variables identified from the set of 97 predictors - 52 donor and 45 recipient characteristics. Hazard ratios of the 12 most prognostic variables for the Cox models are shown in Table 2, based on the absolute z-score values for the Cox model with all variables. The strongest predictor is *re-transplantation*. Having been transplanted before increases the hazard of graft-failure or death by more than 55%. The other most detrimental variables are *donor age* and *donor type circulatory dead*. One unit increase for donor age rises the hazard by around 1% while having received the graft from a donor circulatory versus brain-dead increases the hazard by more than 29% for all models. The rest of the factors which have an adverse effect are: *cold ischemic time*, *diabetes*, *race*, *life-support*, *recipient age*, *incidental tumour*, *spontaneous hypertensive bleeding*, *serology status of HCV* and *intense care unit before the operation*.

In Table 3 the most prognostic factors for the machine learning techniques are presented. The top predictors are provided in terms of relative importance (Rel-Imp) for the PLANN models and in terms of variable importance (VIMP) for the RSF. For the NNs, the strongest predictor is *re-transplantation* (Rel-Imp 0.035 for 1 hidden and 0.028 for 2 hidden layers), which is the second strongest for the RSF (VIMP 0.009). According to the tuned RSF, the most prognostic factor for the overall graft-survival of the patient is *donor age* (VIMP 0.010).

**Table 2:** Hazard ratios along with their 95% confidence intervals for the 12 most influential variables for the Cox models. Variables are presented in decreasing order according to the absolute z-score values (12.90 to 5.16) for the Cox model with all variables. Predictors shown are the most prognostic as their z-scores values correspond to low and very significant *p*-values. These variables were also selected by both Cox backward and Cox LASSO model which verifies their prognostic ability for Cox models

	Cox all variables	Cox backward	Cox LASSO
	HR (95% CI)	HR (95% CI)	HR
Re-transplantation	1.602 (1.491-1.721)	1.608 (1.501-1.722)	1.558
Donor age	1.010 (1.008-1.011)	1.011 (1.009-1.012)	1.009
Donor type DCD ^(*a*)^	1.483 (1.362-1.616)	1.443 (1.338-1.556)	1.298
log(Total cold ischemic time)	1.258 (1.192-1.327)	1.285 (1.221-1.353)	1.191
Diabetes	1.173 (1.125-1.225)	1.176 (1.128-1.226)	1.136
Race Black ^(*b*)^	1.240 (1.171, 1.314)	1.261 (1.193-1.332)	1.186
Life support	1.343 (1.240-1.454)	1.375 (1.272-1.487)	1.304
Recipient age	1.007 (1.005-1.009)	1.008 (1.006-1.010)	1.006
Incidental tumour	1.314 (1.202, 1.437)	1.315 (1.203-1.437)	1.203
Hypertensive bleeding	1.296 (1.185, 1.418)	1.301 (1.190-1.423)	1.214
HCV ^(*c*)^ serology status	1.147 (1.091-1.206)	1.148 (1.094-1.205)	1.166
Pre-treatment status ICU ^(*d*)^	1.240 (1.143, 1.346)	1.253 (1.160-1.354)	1.164

(a): Donor type DCD (Donor Circulatory Dead) vs DBD (Donor after Brain-Dead), (b): Race Black vs White, (c): Chronic hepatitis C virus, (d): Intense Care Unit vs Non-hospitalised/Hospitalised

**Table 3:** The 12 most prognostic factors for the neural networks with 1 and 2 hidden layers (Rel-Imp: relative importance) and for the Random Survival Forest (VIMP: variable importance). Note that the NN utilises time intervals as one of the input variables (check the contribution of time intervals in Table 1 of Additional file 1). For RSF importance is measured for each variable without distinction for each level

Neural network 1h	Rel-Imp	Neural network 2h	Rel-Imp	RSF	VIMP
Re-transplantation	0.035	Re-transplantation	0.028	Donor age	0.010
Life-support	0.025	HCV ^(*d*)^ serology status	0.025	Re-transplantation	0.009
Pre-treatment status ICU ^(*a*)^	0.023	Life-support	0.024	Life support	0.007
Donor type DCD ^(*b*)^	0.023	Donor age	0.023	HCV ^(*d*)^ serology status	0.007
Race Black ^(*c*)^	0.022	Diabetes	0.021	Pre-treatment status	0.006
HCV ^(*d*)^ serology status	0.022	Pre-treatment status ICU ^(*a*)^	0.020	Recipient age	0.004
Diabetes	0.020	Working income	0.020	Aetiology	0.003
Donor age	0.020	Race Black ^(*c*)^	0.019	log(Last serum creatinine)	0.003
Working income	0.018	Previous abdominal surgery	0.015	Functional status	0.002
Functional status Total assistance ^(*e*)^	0.017	Donor pre-recovery diuretics	0.015	log(Total cold ischemic time)	0.002
Aetiology HCV	0.017	Aetiology Cholestatic	0.011	Race	0.002
Hypertensive bleeding	0.017	Functional status Total assistance ^(*e*)^	0.015	Diabetes	0.002

(a): Intense Care Unit vs Non-hospitalised/Hospitalised (b): Donor type DCD (Donor Circulatory Dead) vs DBD (Donor after Brain-Dead), (c): Race Black vs White, (d): Chronic hepatitis C virus, (e): Total assistance vs No assistance

Other strong prognostic variables for the NN with 1 hidden layer are *life support* (Rel-Imp 0.025), *intense care unit before the operation* (Rel-Imp 0.023) and *donor type circulatory dead versus brain-dead* (Rel-Imp 0.023). For the NN with 2 hidden layers other very prognostic variables are *serology status for HCV* (Rel-Imp 0.025), *life support* (Rel-Imp 0.024) and donor age (Rel-Imp 0.023).

For the RSF *life support* (VIMP 0.007), *serology status for HCV* (VIMP 0.007) and *intense care unit before the operation* (VIMP 0.006). Note that variable *total cold ischemic time* which was identified as the 4th most prognostic for the Cox model with all variables and the 10th most prognostic for random survival forest is not in the list of the 12 most prognostic for both NNs.

### Individual predictions

In this section, the predicted survival probabilities are compared for 3 new hypothetical patients and 3 patients from the test data.

**Fig. 2:**
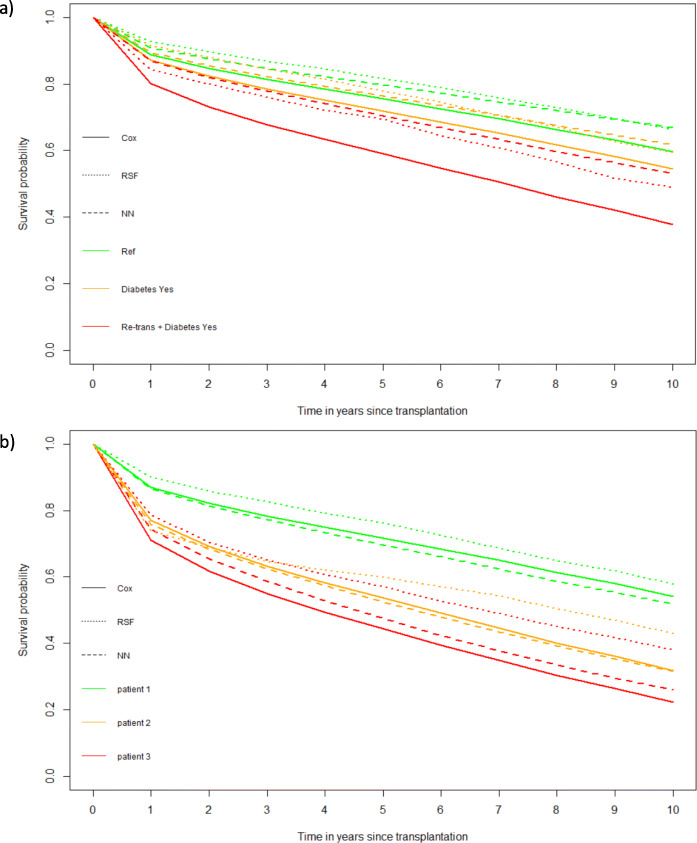
**a** Predicted survival probabilities for 3 new hypothetical patients using the Cox model with all variables (solid lines), the tuned RSF (short dashed lines) and the tuned NN with 1 hidden layer (long dashed lines). The green lines correspond to a reference patient with the median values for the continuous and the mode value for categorical variables. The patient in the orange line has diabetes (the other covariates as in reference patient). The patient in the red line has been transplanted before and has diabetes simultaneously (the other covariates as in reference patient). Values for 10 prognostic variables for the reference patient are provided in Table 2 of Additional file 1. **b** Predicted survival probabilities for 3 patients selected from the test data based on the Cox model with all variables (solid lines), the tuned RSF (short dashed lines) and the tuned NN with 1 hidden layer (long dashed lines). Green lines correspond to a patient censored at 1.12 years. Patient in the orange line was censored at 6.86 years. Patient in the red line died at 0.12 years. Values for 10 prognostic variables for the patients are provided in Tables 3-5 of Additional file 1

In Fig. 2a the patient with reference characteristics shows the best survival. The highest probabilities are predicted by the RSF and the lowest by the Cox model. The same pattern occurs for the patient that suffers from diabetes (orange lines). The patient with diabetes who has been transplanted before has the worst survival predictions. In this case the NN predicts the highest survival probabilities and the Cox model built using all the prognostic factors the lowest.

In Fig. 2b the estimated survival probabilities are showed by the Cox model with all variables, the tuned RSF and the tuned PLANN with 1 hidden layer for 3 patients from the test set. The first patient shows the highest survival predictions by the 3 models. The RSF provides the highest survival probabilities and the NN the lowest. The second patient experiences lower survival probabilities (orange lines) whereas the third patient shows the lowest survival probabilities overall. For the second patient the NN predicts the lowest survival probabilities over time and for the third the Cox model.

In general, the random survival forest provides the most optimistic survival probabilities whereas the most pessimistic survival probabilities are predicted by either the Cox model or the NN (more often by the Cox model). This may be related to the characteristics of the methods as RSF relies on recursive binary partitioning of predictors, whereas Cox models imply linearity, and NNs fit non-linear relationships.

### Calibration

Here 4 methods are compared: Cox model with all variables, RSF, PLANN 1 hidden and 2 hidden layers based on the calibration on the test data. For each method, the predicted survival probabilities at each year are estimated and the patient data are split into 10 equally sized groups based on the deciles of the probabilities. Then the survival probabilities along with their 95% confidence intervals are calculated using the Kaplan-Meier methodology [47].

**Fig. 3:**
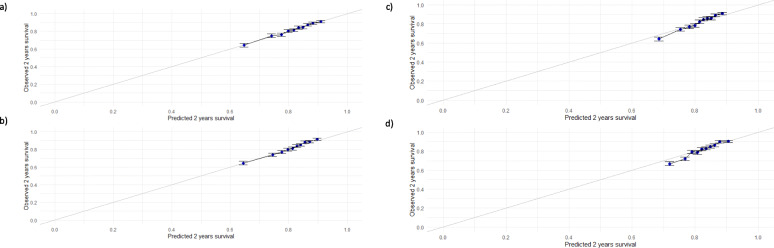
Calibration plots at 2 years on the test data: **a** Cox model with all variables, **b** Random Survival Forest, **c** Partial Logistic Artificial Neural Network with 1 hidden layer, **d** Partial Logistic Artificial Neural Network with 2 hidden layers

In Fig. 3 the results are showed at 2 years since LT. The Cox model with all variables and the PLANN with 1 hidden layer are both well calibrated. The RSF and the PLANN with 2 hidden layers tend to overestimate the survival probabilities for the patients at higher risk. Survival neural network with 1 hidden layer seems to be the most reliable for predictions between the ML techniques. Calibration plots at 5 and 10 years can be found in Additional file 3.

## Discussion

With the rise of computational power and technology on the 21 ^*st*^ century, more and more data have been collected in the medical field to identify trends and patterns which will allow building better allocation systems for patients, provide more accurate prognosis and diagnosis as well as more accurate identification of risk factors. During the past few years, machine learning (ML) has received increased attention in the medical area. For instance, in the area of LTs graft failure or primary non-function might be predicted at decision time with ML methodology [48]. Briceño et al. created a NN process for donor-recipient matching specifying a binary classification survival output (recipient or graft survival) to predict 3-month graft mortality [49].

In this study statistical and ML models were estimated for patients from the US post-transplantation. Random survival forest performed better than Cox models with respect to the C-index. This shows the ability of the model to discriminate between low and high risk groups of patients. The C-index was not estimated for NN because a natural ordering of subjects is not feasible. Therefore, the Brier score was measured each year for all methods. The RSF showed similar results to the Cox models having slightly smaller total prediction error (in terms of IBS). The NNs performed in general better than the Cox models or the RSF and had very similar performance over time. RSF and survival NN are ML techniques which have a different learning method and model non-linear relationships between variables automatically. Both methods may be used in medical application but should be applied at present as additional analysis for comparison.

Special emphasis was given on the interpretation of the models. An indirect comparison was performed to examine which are the most prognostic variables for a Cox model with all variables, a RSF and NNs. Results showed that Cox model with all variables (via absolute z-score values) and the NNs with one/two hidden layer(s) (via relative importance) identified similar predictors. Both methods identified *re-transplantation* as the strongest predictor and *donor age*, *diabetes*, *life support* and *race* as relatively strong predictors. According to RSF, the most prognostic variables were *donor age*, *re-transplantation*, *life support* and *serology status of HCV*. *Aetiology* and *last serum creatinine* were selected as the 7^*th*^ and the 8^*th*^ most prognostic. This raises a known concern about the RSF bias towards continuous variables and categorical variables with multiple levels [50] (*aetiology* has 9 levels: metabolic, acute, alcoholic, cholestatic, HBV, HCV, malignant, other cirrhosis, other unknown). As continuous and multilevel variables incorporate larger amount of information than categorical, they tend to be favoured by the splitting rule of the forest during binary partitioning. Such bias was reflected in the variable importance results.

When comparing statistical models with machine learning techniques with respect to interpretability, Cox models offer a straightforward interpretation through the hazard ratios. On the contrary, for both neural networks and random survival forests the sign of the prediction is not provided (if the effect is positive or negative). Additionally, for NNs interpretation is possible for different variable levels (with the method of Garson and its extension), whereas for RSF only the total effect of a variable is shown. There is no common metric to directly compare Cox models with ML techniques in terms of interpretation. Future research in this direction is needed.

ML techniques are inherently based on mechanisms introducing randomisation and therefore very small changes are expected between different iterations of the same algorithm. To evaluate stability of performance, ML models were run several times under the same parametrisation. RSF were consistently stable after a certain number of trees (300 were selected). This was not the case for the NNs where instability is a common problem. It is challenging to tune a NN due to many hyper-parameter combinations available and the lack of a consistent global performance measure for survival data. IBS was used to tune the novel NNs, which may be the reason of instability for the NN with 2 hidden layers together with the large number of weights. Note also that the NN with 1 hidden layer is well calibrated whereas the NN with 2 hidden layers is less calibrated on the test data.

This is the first study where ML techniques are applied to transplant data where a comparison with the traditional Cox model was investigated. To construct the survival NN, the original problem had to be converted into a classification problem where exact survival times were transformed into (maximum) 10 time intervals denoting years since transplantation. On the other hand, for the Cox models and the RSF exact time to event was used. Recently, a new feed forward NN has been proposed for omics data which calculates directly a proportional hazards model as part of the output node using exact time information [51]. A survival NN with exact times may lead to better predictive performance. For UNOS data, 69.1% of the recipients were alive/censored and 30.9% had the event of interest. Results above were based on these particular percentages for censoring and events (for the NNs the percentages varied because of the reformulation of the problem).

It might be useful to investigate how the number of variables affects the performance of the models. Here 97 variables were pre-selected supported by clinical and statistical reasons (e.g. variables available before or during LT). It might be interesting to repeat the analyses on a smaller group of predictors, implementation time can be drastically reduced as the calculation complexity depends on sample size and predictors multiplicity. Alongside, predictive accuracy might be increased as some noisy factors will be removed from the dataset increasing the signal of potentially prognostic variables.

Both traditional Cox models and PLANNs allow for the inclusion of time-dependent covariates. For PLANNs, each patient is replicated multiple times during the transformation of exact times into a set of *k* non-overlapping intervals in long format. Thus, different values of a covariate can be naturally incorporated to increase the predictive ability of the networks. It would be interesting to apply and compare the predictive ability of time-dependent Cox models and PLANNs to liver transplantation data including explanatory variables whose values change over time. Such extension to more dynamic methods may increase predictive performance and help in decision making.

## Conclusions

There is an increased attention to ML techniques beyond SM in the medical field with methods and applications being more necessary than ever. Utilization of these algorithmic approaches can lead to pattern discovery in the data promoting fast and accurate decision making. For time-to-event data, more ML techniques may be applied for prediction such as Support Vector Machines and Bayesian Networks. Moreover, deep learning with NN is gaining more and more attention and will likely be another trend in the future for these complex data.

In this work two alternatives to the Cox model from machine learning for medical data with large total sample size (62294 patients) and many predictors (97 in total) were discussed. RSF showed better performance than the Cox models with respect to C-index so it can be a useful tool for prioritisation of particular high risk patients. NNs showed better prediction performance in terms of Integrated Brier score. However, both ML techniques required a non-trivial implementation time. Cox models are preferable in terms of straightforward interpretation and fast implementation. Our study suggests that some caution is required when ML methods are applied to survival data. Both approaches can be used for exploratory and analysis purposes as long as the advantages and the disadvantages of the methods are presented.

## Supplementary Information

**Additional file 1** Includes the Garson’s algorithm for 2 hidden layers, a table with the relative importance of the time intervals for the neural networks with 1 and 2 hidden layes, detailed criteria for variable pre-selection, a plot of survival and censoring distributions and 4 tables with individual patient characteristics.

**Additional file 2** Provides information about the package to implement RSFs and NNs as well as technical parts regarding the choice of tuning parameters and the cross-validation procedure for each method. A figure illustrates the cross-validation procedure for RSF on a 3D space. References are provided for further reading.

**Additional file 3** Contains calibration plots at 5 and 10 years for a) a Cox model with all prognostic factors, b) a Random Survival Forest with all prognostic factors, c) a Partial Logistic Artificial Neural Network with 1 hidden layer with all prognostic factors and d) a Partial Logistic Artificial Neural Network with 2 hidden layers with all prognostic factors.

**Additional file 4** Provides the R code developed for the analyses of this project.

## Data Availability

The research data for this project is private. Unauthorized use is a violation of the terms of the Data Use Agreement with the U.S. Department of Health and Human Services. More information and instructions for researchers to request UNOS data can be found at https://unos.org/data/. R-code developed to perform the analysis is available in Additional file 4.

## Abbreviations

BS: Brier score
CVPL: cross-validated log-partial likelihood
DCD: Donor Circulatory Dead
HBV: Chronic hepatitis B virus
HCV: Chronic hepatitis C virus
IBS: Integrated Brier score
IPCW: Inverse Probability of Censoring Weighting
LASSO: least angle and selection operator
LT: liver transplantation
LUMC: Leiden University Medical Center
ML: machine learning
NN(s): artificial neural network(s)
OGS: overall graft-survival
OPO: Organ Procurement Organisations
OPTN: Organ Procurement and Transplantation Network
PLANN: partial logistic artificial neural network
PLANN-ARD: partial logistic artificial neural network - automatic relevance determination
PH: proportional hazards
Rel-Imp: relative importance
RSF: random survival forest
SM: statistical model
SRTR: Scientific Registry of Transplant Recipients
UNOS: United Network of Organ Sharing
VIMP: variable importance.

## Footnotes

1 UNOS is a non-profit and scientific organisation in the United States which arranges organ donation and transplantation. For more information visit its website https://unos.org.

2 Dictionary for variables details is provided at: https://www.srtr.org/requesting-srtr-data/saf-data-dictionary/.
