# Supplementary material for: Survival prediction models since liver transplantation - comparisons between Cox models and machine learning techniques
Source: BMC Med Res Methodol. 2020 Nov 16;20:277. doi: 10.1186/s12874-020-01153-1 (PMC7667810; doi:10.1186/s12874-020-01153-1)
Supplement: Supplementary file 1 — Additional file 1 Includes the Garson’s algorithm for 2 hidden layers, a table with the relative importance of the time intervals for the neural networks with 1 and 2 hidden layes, detailed criteria for variable pre-selection, a plot of survival and censoring distributions and 4 tables with individual patient characteristics. [file 12874_2020_1153_MOESM1_ESM.pdf]

# Supplementary material

## Garson's algorithm for 2 hidden layers

**Algorithm 1:** Garson weights algorithm - 2 hidden layers

**Input:** connection weight matrices $W_1$, $W_2$, $W_3$ for input-hidden1, hidden1-hidden2, hidden2-output layers of dimensions ($\alpha \times \alpha_1$), ($\alpha_1 \times \alpha_2$), ($\alpha_2 \times 1$) respectively, where $\alpha$ is the number of the input nodes, $\alpha_1$ is the number of the nodes in hidden layer 1 and $\alpha_2$ the number of nodes in hidden layer 2

**Output:** relative importance $R_j$

$W = (W_1 W_2 W_3)^T$ of dimensions ($1 \times \alpha$)

$W^* = |W|$

$R_j = \frac{W^*_{1j}}{\sum_{j=1}^{p} W^*_{1j}}, \ \forall j \in (1, 2, \cdots, \alpha)$

## Relative importance for each time interval

| Neural network 1h | Rel-Imp. | Neural network 2h | Rel-Imp. |
|---|---|---|---|
| Interval 1 | 0.125 | Interval 1 | 0.128 |
| Interval 4 | 0.025 | Interval 4 | 0.029 |
| Interval 5 | 0.022 | Interval 3 | 0.027 |
| Interval 3 | 0.020 | Interval 5 | 0.025 |
| Interval 6 | 0.017 | Interval 6 | 0.018 |
| Interval 7 | 0.011 | Interval 7 | 0.014 |
| Interval 2 | 0.004 | Interval 2 | 0.010 |
| Interval 8 | 0.004 | Interval 9 | 0.006 |
| Interval 9 | 0.003 | Interval 8 | 0.005 |
| Interval 10 | 0.003 | Interval 10 | 0.003 |

Table 1: Relative importance for each of the 10 time intervals for the neural networks (training set).

## Criteria for variable pre-selection

The data provided by UNOS included 62294 patients who underwent liver transplant surgery between 2005 and 2015. Standard analysis files contained 657 variables regarding donors and patients (candidates and recipients). These regarded:

- **identification variables** like *unique encrypted person id*, *unique encrypted donor id*, *candidate listing center*, *OPO serving transplant center*.
- **important dates** such as *transplant date*, *graft failure date*, *cohort censoring date*, *death date*, *graft follow-up date*.
- **status variables**: *death status* (in 1, 3, 5 years and later), *graft failure-free status* (in 1, 3, 5 years and later).
- **demographic variables** such as *age*, *gender*, *race*, *ethnicity*, *socioeconomic status* and *education level*.
- **behavioral variables** e.g. *smoking history*, *alcohol consumption*, *physical activity level*, *cocaine or other drug history*.
- **physiological variables** for example *blood type*, *etiology* (cause of disease), *laboratory measurements* for arginine, serum creatinine, serum sodium, total bilirubin etc.

From those, 97 risk factors (52 donor, 45 patient characteristics) were pre-selected. Our variable pre-selection was based on the following **clinical** and **statistical** grounds:

1. Clinical importance of particular prognostic factors in bibliography regrading LT.
2. Experts in LT from Leiden University Medical Centre (LUMC).
3. Variables available after performing LT were discarded.
4. Variables with more than 40% missing values were excluded. To explain this, it would be infeasible to reconstruct variables with more than 40% missingness with plausible values based on the distribution of the observed data, as they would most likely be noisy factors.
5. Categorical variables with very unbalanced classes (less than 1% sample size for a level) were dropped as they could pose a serious threat to the modelling procedure.
6. Redundant variables were discarded (e.g. *age of the donor* in years and in months, or a numerical variable with one value, or a categorical variables with a single level).

For patients, there were several variables referring to both candidates and recipients as for instance *last encephalopathy*, *diabetes* or *hypertension status*. From those, we pre-selected the ones corresponding to the recipients as they were more relevant for this project. Keeping the variables for the candidates would be more relevant if the focus was on the waiting list mortality.

## Individual characteristics

| Variable | Value | Variable | Value |
|---|---|---|---|
| Donor age | 42 | On life support | ’No’ |
| Donor type | ’Donor Brain Dead’ | Pre-treatment status | ’Not hospitalised’ |
| Diabetes | ’No’ | Race | ’White’ |
| HCV serology status | ’No’ | Recipient age | 56 |
| log(Total cold ischemic time) | 1.99 | Re-transplantation | ’No’ |

Table 2: Values for 10 potentially prognostic variables of the reference patient according to our models sorted in alphabetical order. The patient was constructed using the median values for the continuous and the mode values for categorical variables. Patient characteristics were obtained from the test data.

| Variable | Value | Variable | Value |
|---|---|---|---|
| Donor age | 39 | On life support | ’Yes’ |
| Donor type | ’Donor Brain Dead’ | Pre-treatment status | ’Intense Care Unit’ |
| Diabetes | ’No’ | Race | ’White’ |
| HCV serology status | ’Yes’ | Recipient age | 44 |
| log(Total cold ischemic time) | 2.09 | Re-transplantation | ’No’ |

Table 3: Values for 10 potentially prognostic variables of a patient censored at 1.12 years according to our models sorted in alphabetical order. Patient characteristics were obtained from the test data.

| Variable | Value | Variable | Value |
|---|---|---|---|
| Donor age | 68 | On life support | ’No’ |
| Donor type | ’Donor Brain Dead’ | Pre-treatment status | ’Not hospitalised’ |
| Diabetes | ’Yes’ | Race | ’White’ |
| HCV serology status | ’No’ | Recipient age | 61 |
| log(Total cold ischemic time) | 2.08 | Re-transplantation | ’No’ |

Table 4: Values for 10 potentially prognostic variables of a patient censored at 6.86 years according to our models sorted in alphabetical order. Patient characteristics were obtained from the test data.

| Variable | Value | Variable | Value |
|---|---|---|---|
| Donor age | 54 | On life support | ’No’ |
| Donor type | ’Donor Brain Dead’ | Pre-treatment status | ’Not hospitalised’ |
| Diabetes | ’No’ | Race | ’White’ |
| HCV serology status | ’Yes’ | Recipient age | 60 |
| log(Total cold ischemic time) | 2.08 | Re-transplantation | ’No’ |

Table 5: Values for 10 potentially prognostic variables of a patient who died at 0.12 years according to our models sorted in alphabetical order. Patient characteristics were obtained from the test data.

# Survival and censoring distributions

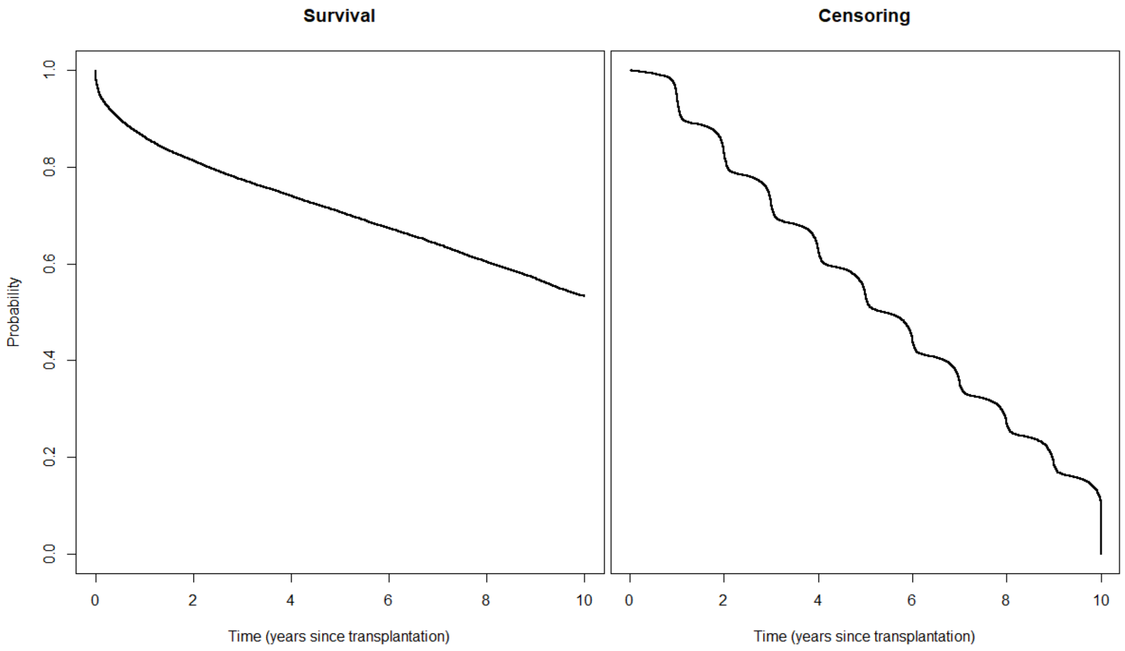


Figure 1: Survival and censoring distribution for the 41530 patients of the training data (overall graft-survival).
