# Supplementary material for: Survival prediction models since liver transplantation - comparisons between Cox models and machine learning techniques
Source: BMC Med Res Methodol. 2020 Nov 16;20:277. doi: 10.1186/s12874-020-01153-1 (PMC7667810; doi:10.1186/s12874-020-01153-1)
Supplement: Supplementary file 2 — Additional file 2 Provides information about the package to implement RSFs and NNs as well as technical parts regarding the choice of tuning parameters and the cross-validation procedure for each method. A figure illustrates the cross-validation procedure for RSF on a 3D space. References are provided for further reading. [file 12874_2020_1153_MOESM2_ESM.pdf]

Survival prediction models since liver transplantation - comparisons between Cox models and machine learning techniques

Georgios Kantidakis[a,b,c,*], Hein Putter[b], Carlo Lancia[a], Jacob de Boer[d], Andries E Braat[d], Marta Fiocco[a,b,e]

[a]*Mathematical Institute Leiden University, Niels Bohrweg 1, 2333 CA Leiden, The Netherlands*

[b]*Department of Biomedical Data Sciences, Section Medical Statistics, Leiden University Medical Center (LUMC), Albinusdreef 2, 2333 ZA Leiden, The Netherlands*

[c]*Department of Statistics, European Organisation for Research and Treatment of Cancer (EORTC) Headquarters, Ave E. Mounier 83/11, 1200 Brussels, Belgium*

[d]*Department of Surgery, Leiden University Medical Center (LUMC), Albinusdreef 2, 2333 ZA Leiden, The Netherlands*

[e]*Trial and Data Center, Princess Máxima Center for pediatric oncology (PMC), Heidelberglaan 25, 3584 CS Utrecht, The Netherlands*

[*]*Corresponding author, emails: G.Kantidakis@lumc.nl, georgios.kantidakis@eortc.org*

Model training

The split sample approach was employed; data was split randomly into two complementary parts, a training set (2/3) and a test set (1/3) under the same event/censoring proportions. To tune a model, 5-fold cross validation was performed in the training set for the machine learning techniques (and for Cox LASSO). Training data was divided into 5 folds. Each time 4 folds were used to train a model and the remaining fold was used to validate its performance and the procedure was repeated for all combination of folds. Tuning of the hyper-parameters was done using grid search and performance of final models was assessed on the test set.

For RSF the `randomForestSRC` package [1] of the R programming language was used. The choice of the hyper-parameters and their range (grid search) was provided based on recommendations by the

authors of `randomForestSRC` [2]. Parameters tuned were `ntree` the number of bootstrapped trees grown (range 1-500), `mtry` the number of candidate variables examined at each split point (range 5-47), `nsplit` the number of split points at which an X-variable is tested using the log-rank splitting rule (range 3-7) [3] and `nodesize` (range 10-100) the average number of observations in the terminal nodes across the forest. In general, parameters `ntree` and `mtry` are the most fundamental for RSF. The parameter `ntree` modulates the consistency of the forest's performance and `mtry` controls an important part of randomness during the growth of decision trees. Parameter `nsplit` with `nsplit` $>$ 0 can be used to trigger a randomised selection of exactly nsplit points for each of the mtry variables within a node h. Last, parameter `nodesize` plays an important role in the topology of the trees as it controls the average node size of the forest. Large values in `nodesize` parameter will essentially force the forest to under-grow whereas small values will lead each tree to keep growing on with more and more noisy variables being selected. The best combination of the parameters was determined based on the error of the forests defined as $E = 1 - C$, where C is Harrell's concordance index [4] based on the ensemble survival mortality of individuals. Figure 1 shows the cross-validated error E for different values of tuning parameters `mtry`, `nodesize` and `nsplit`. Optimal values were given by `mtry` = 12, `nodesize` = 50 and `nsplit` = 5.

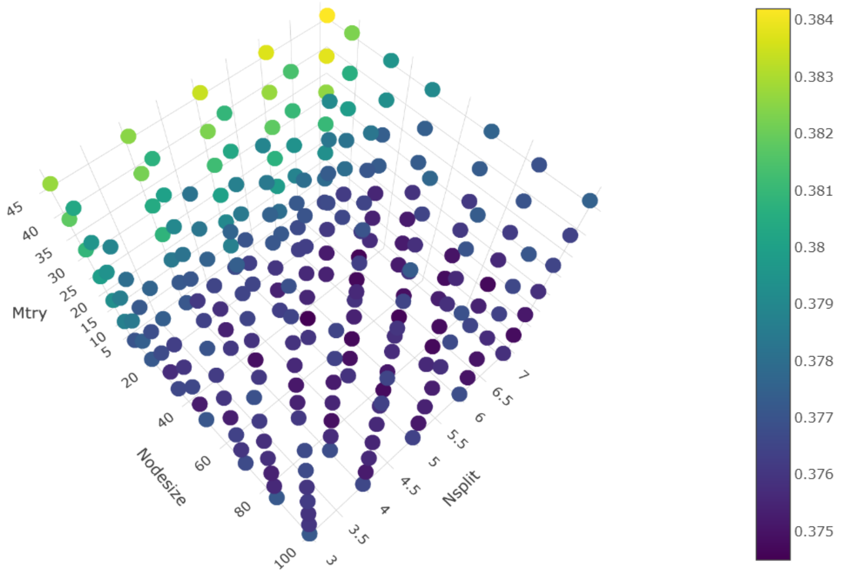

Figure 1: Cross-validation applied on a 3-D space for the RSF. Parameters tuned are the number of candidate variables (`mtry`), the average node size across the forest (`nodesize`) and the number of split points for each x-variable (`nsplit`). The bar on the right side of the graph shows the **error E** corresponding to combinations on training data. Dark blue dots correspond to lower prediction error E and represent the best hyper-parameter combinations whereas green and yellow dots correspond to poorly performed combinations.

For survival NNs, standard software of implementation was not available in R. Therefore, data transformation to longitudinal format was required. The task was turned into a classification problem where patients were divided into maximum 10 time intervals on the training set and exactly 10 intervals on the validation set (training data) and test set (new unseen data) and the intervals were added to the other features as covariates to estimate conditional hazard probabilities. This led to a training set of 194635, a validation set of 415300 (training data of 41530 patients replicated for 10 intervals), and a test set of

207640 observations in long format (test data duplicated for 10 intervals). Variables were presented in dummy coding and all continuous factors were standardized. Model tuning was performed in R with the package `keras` [5] which is an interface for the original state-of-the-art NN library written in Python programming language. `keras` runs on top of `tensorflow` [6] which is a symbolic maths library used for machine learning. Two of the main advantages of the package are that it allows the use of distributed training of deep learning models on clusters of graphic processing units and the specification of many building blocks such as layers, objectives, activation functions, optimisers.

To narrow down the grid of point combinations, search for training data was performed on a 5-D space of some of the most fundamental hyper-parameters for the ML community. Those are `nodesize` the size of nodes in the hidden layer(s) (range 10-130), `dropout rate` that randomly selects the amount of nodes to be dropped-out with a given probability (0.1, 0.2 or 0.3), `learning rate` which is the step size of weight iteration (0.01, 0.1 or 0.2), `momentum` which helps to accelerate gradient vectors (0.8 or 0.9) and `weak class weight` that defines the weight of minority class (1, 2.236 or 10). `nodesize` defines the number of weights of the network and consequently the amount of its complexity. Having 129 inputs in total (119 potentially prognostic variable levels in dummy coding + 10 time intervals), the optimum node size parameter will be somewhere in the range 10 - 130. `Dropout rate` is a technique used to control over-fitting [7]. Additionally, we used early stopping to prevent over-fitting (5 epochs tolerance). We specified 50 training epochs (an arbitrary large number) and terminated models' training once the performance stopped improving on the validation set. Regarding the rest of the parameters, `learning rate` adjusts how fast the stochastic gradient descent iterative method uses stochastic approximation. `Momentum` can accelerate the stochastic gradient vectors in the right directions and `weak class weight` can be used for re-weighting unbalanced classes. In this dataset, 30.9 % of the patients experienced the event of interest so it was investigated if the performance can be improved by re-weighting the minority class with 2.236 (= 69.1 / 30.9). Moreover, in the long data format each patient was replicated for a maximum of 10 intervals on the training data and for exactly 10 intervals on the validation data, it was investigated if the performance can be improved by re-weighting 10 times the minority class. We restrict ourselves to these 5 hyperparameters: `nodesize`,`dropout rate`, `learning rate`, `momentum` and `weak class weight` to avoid a massive amount of grid search combinations by tuning more parameters (e.g batch size).

For survival NNs there is no well-established measure of cross-validation performance as they have rarely been used in practice. The best combinations of hyper-parameters were selected based on the Integrated Brier Score (IBS) - a measure that summarises the time-dependent Brier score estimated at different time points until 10 years in one value. Common hyperparameters which play an important role in the performance of the models in `keras` were selected that satisfy necessary constraints and can generalise different data patterns. Afterwards, the search was repeated on a narrower domain centred around highly performing combinations.

References

[1] Kogalur U and Ishwaran H. randomForestSRC: Fast unified random forests for survival, regression, and classification (RF-SRC). *R package version*, 2(1), 2019.

[2] Random Forests for Survival, Regression, and Classification. URL: https://kogalur.github.io/randomForestSRC/theory.html.

[3] Mark Robert Segal. Regression Trees for Censored Data. *Biometrics*, 44(1):35–47, 1988. URL: http://www.jstor.org/stable/2531894.

[4] J. C. van. Houwelingen and Hein. Putter. *Dynamic prediction in clinical survival analysis*. CRC Press, 2012. URL: https://www.crcpress.com/Dynamic-Prediction-in-Clinical-Survival-Analysis/van-Houwelingen-Putter/p/book/9781439835333.

[5] JJ Allaire and François Chollet. keras: R Interface to 'Keras', 2018. URL: https://CRAN.R-project.org/package=keras.

[6] Martin Abadi, Paul Barham, Jianmin Chen, Zhifeng Chen, Andy Davis, Jeffrey Dean, Matthieu Devin, Sanjay Ghemawat, Geoffrey Irving, Michael Isard, Manjunath Kudlur, Josh Levenberg, Rajat Monga, Sherry Moore, Derek G. Murray, Benoit Steiner, Paul Tucker, Vijay Vasudevan, Pete Warden, Martin Wicke, Yuan Yu, and Xiaoqiang Zheng. TensorFlow: A system for large-scale machine learning. In *12th {USENIX} Symposium on Operating Systems Design and Implementation ({OSDI} 16)*, pages 265–283, 2016. URL: https://ai.google/research/pubs/pub45381.

[7] Nitish Srivastava, Geoffrey Hinton, Alex Krizhevsky, Ilya Sutskever, and Ruslan Salakhutdinov. Dropout: A Simple Way to Prevent Neural Networks from Overfitting. *Journal of Machine Learning Research*, 15(1):1929–1958, 2014. URL: http://jmlr.org/papers/v15/srivastava14a.html.
