# Supplementary material for: Survival prediction models since liver transplantation - comparisons between Cox models and machine learning techniques
Source: BMC Med Res Methodol. 2020 Nov 16;20:277. doi: 10.1186/s12874-020-01153-1 (PMC7667810; doi:10.1186/s12874-020-01153-1)
Supplement: Supplementary file 3 — Additional file 3 Contains calibration plots at 5 and 10 years for a) a Cox model with all prognostic factors, b) a Random Survival Forest with all prognostic factors, c) a Partial Logistic Artificial Neural Network with 1 hidden layer with all prognostic factors and d) a Partial Logistic Artificial Neural Network with 2 hidden layers with all prognostic factors. [file 12874_2020_1153_MOESM3_ESM.docx]

**Calibration plots at 5 years**

1. **Cox model**

**
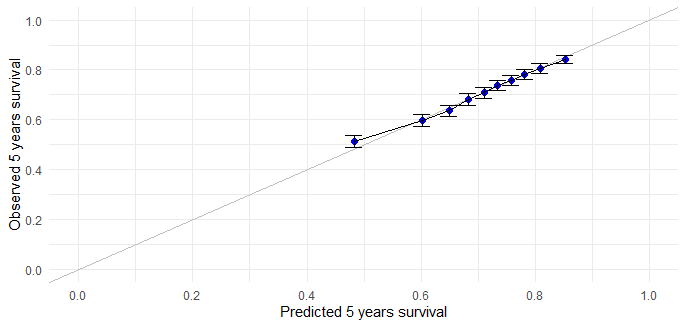
**

1. **Random Survival Forest**

**
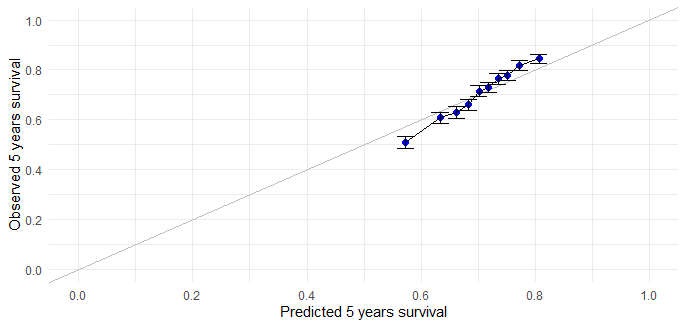
**

1. **Partial Logistic Artificial Neural Network (1 hidden layer)**

**
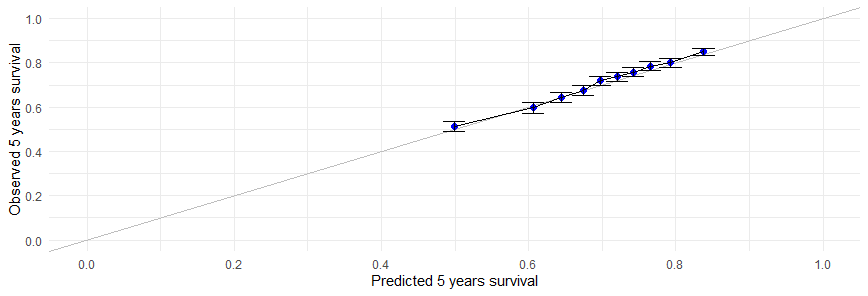
**

1. **Partial Logistic Artificial Neural Network (2 hidden layers)**

**
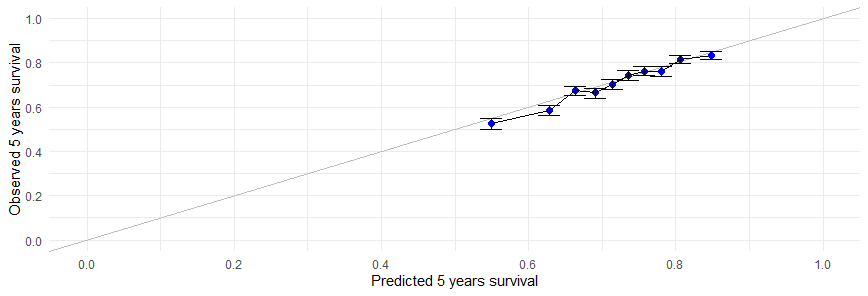
**

**Calibration plots at 10 years**

1. **Cox model**

**
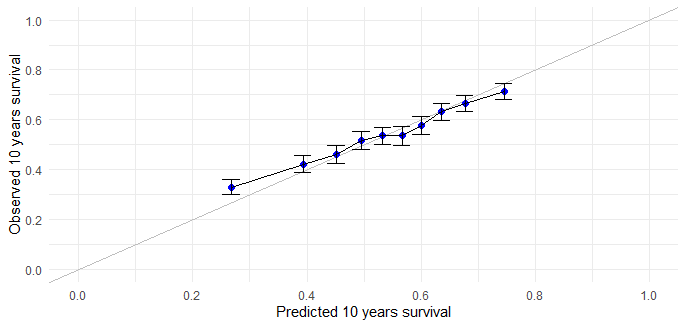
**

1. **Random Survival Forest**

**
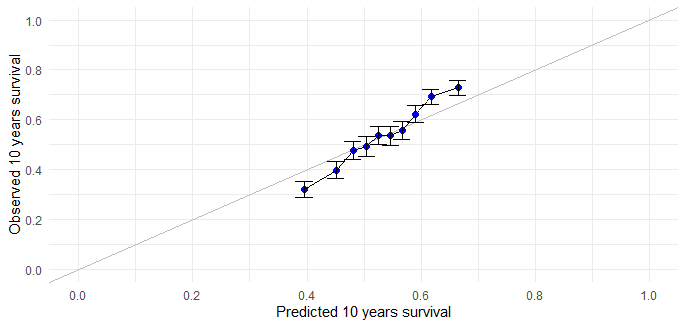
**

1. **Partial Logistic Artificial Neural Network (1 hidden layer)**

**
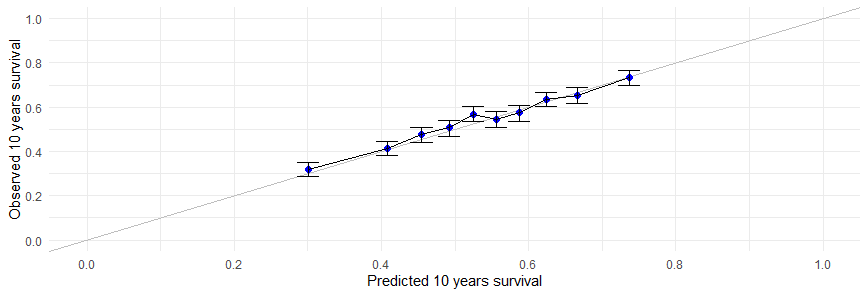
**

1. **Partial Logistic Artificial Neural Network (2 hidden layers)**

**
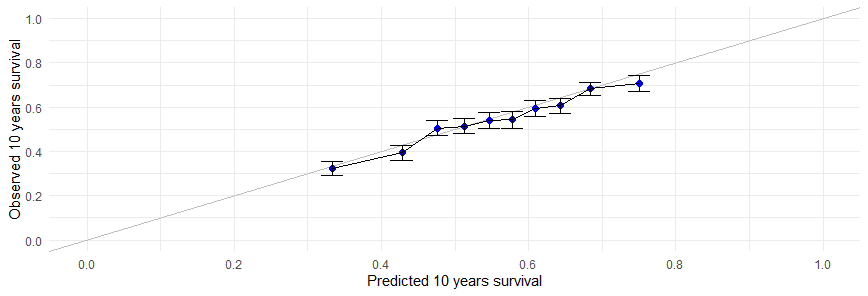
**
