# Supplementary material for: Survival prediction models since liver transplantation - comparisons between Cox models and machine learning techniques
Source: BMC Med Res Methodol. 2020 Nov 16;20:277. doi: 10.1186/s12874-020-01153-1 (PMC7667810; doi:10.1186/s12874-020-01153-1)
Supplement: Supplementary file 4 — Additional file 4 Provides the R code developed for the analyses of this project. [file 12874_2020_1153_MOESM4_ESM.docx]

Project: **Survival prediction models since liver transplantation**

R-code for the comparison between Cox models and machine-learning techniques.

The Cox models used are:

1. A cox model with 97 prognostic factors (52 regarding donor and 45 regarding patient characteristics)
2. A Cox model with backward elimination
3. A Cox model with LASSO selection

The machine learning techniques used are:

a) A random survival forest (RSF)

b) A partial logistic artificial neural network (PLANN) with 1 hidden layer

c) A partial logistic artificial neural network (PLANN) with 2 hidden layers

####################################################################

# Data imputation

####################################################################

install_packages <- c("caret", "dplyr", "Hmisc", "randomForestSRC")

for (i in 1:length (install_packages)){

if (!install_packages[i] %in% installed.packages()){

install.packages(install_packages[i])

}

}

library(caret)

library(dplyr)

library(Hmisc)

library(randomForestSRC)

library(parallel) # is a base library

options(rf.cores = 20, mc.cores = 20) # to use 20 cores on parallel for randomForestSRC

load("unos_selected.RData")

levels(unos_selected$etiology)[levels(unos_selected$etiology) == "Metabolic (ref)"] <- "Metabolic"

unos_selected$etiology <- relevel(unos_selected$etiology, ref = "Metabolic")

unos_selected <- unos_selected %>% mutate_if(is.factor, funs(`levels<-`(., trimws(levels(.)))))

unos_selected <- unos_selected %>% mutate_if(is.factor,

funs(`levels<-`(., gsub("/", "_or_", levels(.)))))

unos_selected <- unos_selected %>% mutate_if(is.factor,

funs(`levels<-`(., gsub("-", "_to_", levels(.)))))

unos_selected <- unos_selected %>% mutate_if(is.factor,

funs(`levels<-`(., gsub("<", "smaller", levels(.)))))

unos_selected <- unos_selected %>% mutate_if(is.factor,

funs(`levels<-`(., gsub(">=", "geq", levels(.)))))

unos_selected <- unos_selected %>% mutate_if(is.factor,

funs(`levels<-`(., gsub(" ", "_", levels(.)))))

# table(unos_selected$etiology)

# str(unos_selected$etiology)

# relevel some variables

unos_selected$donorrace <- relevel(unos_selected$donorrace, ref = "White")

unos_selected$don_txfus_terminal_hosp_num <- relevel(unos_selected$don_txfus_terminal_hosp_num,

ref = "None")

unos_selected$albumin2 <- relevel(unos_selected$albumin2, ref = "geq2")

unos_selected$split <- relevel(unos_selected$split, ref = "whole_organ")

unos_selected$lifesupport <- relevel(unos_selected$lifesupport, ref = "No_lifesupport")

unos_selected$pretxstatus <- relevel(unos_selected$pretxstatus, ref = "NOT_HOSPITALIZED")

unos_selected$can_race_srtr <- relevel(unos_selected$can_race_srtr, ref = "White")

unos_selected$can_education <- relevel(unos_selected$can_education,

ref = "None_or_low_or_undefined_education")

save(unos_selected, file = "unos_selected.RData")

# check how many variables are continuous

which(sapply(unos_selected[1:97], is.numeric))

# check how many variables are factors

sum(sapply(unos_selected[1:97], is.factor))

# re-arrange some continuous variables

#check the distribution of continuous variables

hist(unos_selected$donorage); summary(unos_selected$donorage) # OK

hist(unos_selected$don_hgt_cm); summary(unos_selected$don_hgt_cm) # OK

hist(unos_selected$don_wgt_kg); summary(unos_selected$don_wgt_kg) # OK

hist(unos_selected$don_bmi); summary(unos_selected$don_bmi) # OK

hist(unos_selected$don_log_sgot); summary(unos_selected$don_log_sgot) # OK

hist(unos_selected$don_creat); summary(unos_selected$don_creat) # probl

hist(unos_selected$don_log_bun); summary(unos_selected$don_log_bun) # OK

hist(unos_selected$don_tot_bili); summary(unos_selected$don_tot_bili) # probl

hist(unos_selected$don_log_sgpt); summary(unos_selected$don_log_sgpt) # Ok

hist(unos_selected$don_sodium); summary(unos_selected$don_sodium) # OK

hist(unos_selected$don_inr); summary(unos_selected$don_inr) # probl

hist(unos_selected$don_hematocrit); summary(unos_selected$don_hematocrit) # Ok

hist(unos_selected$recipientage); summary(unos_selected$recipientage) # OK

hist(unos_selected$coldischemiatime); summary(unos_selected$coldischemiatime) # probl

hist(unos_selected$rec_bmi); summary(unos_selected$rec_bmi) # Ok

hist(unos_selected$can_last_bili); summary(unos_selected$can_last_bili) #probl

hist(unos_selected$can_last_inr); summary(unos_selected$can_last_inr) # probl

hist(unos_selected$can_last_serum_creat); summary(unos_selected$can_last_serum_creat) # probl

hist(unos_selected$can_last_serum_sodium); summary(unos_selected$can_last_serum_sodium) # Ok

hist(unos_selected$rec_hgt_cm); summary(unos_selected$rec_hgt_cm) # OK

hist(unos_selected$rec_wgt_kg); summary(unos_selected$rec_wgt_kg) # OK

# transform the 7 variables that are not normally distributed

summary(unos_selected$don_creat)

unos_selected$don_creat <- log(unos_selected$don_creat)

which(colnames(unos_selected) == "don_creat")

colnames(unos_selected)[12] <- "don_logcreat"

summary(unos_selected$don_tot_bili)

unos_selected$don_tot_bili <- log(unos_selected$don_tot_bili)

which(colnames(unos_selected) == "don_tot_bili")

colnames(unos_selected)[27] <- "don_logtot_bili"

summary(unos_selected$don_inr)

unos_selected$don_inr <- log(unos_selected$don_inr)

which(colnames(unos_selected) == "don_inr")

colnames(unos_selected)[31] <- "don_loginr"

summary(unos_selected$coldischemiatime)

unos_selected$coldischemiatime <- log(unos_selected$coldischemiatime + 1)

which(colnames(unos_selected) == "coldischemiatime")

colnames(unos_selected)[68] <- "log_coldischemiatime"

summary(unos_selected$can_last_bili)

unos_selected$can_last_bili <- log(unos_selected$can_last_bili)

which(colnames(unos_selected) == "can_last_bili")

colnames(unos_selected)[86] <- "can_last_logbili"

summary(unos_selected$can_last_inr)

unos_selected$can_last_inr <- log(unos_selected$can_last_inr)

which(colnames(unos_selected) == "can_last_inr")

colnames(unos_selected)[87] <- "can_last_loginr"

summary(unos_selected$can_last_serum_creat)

unos_selected$can_last_serum_creat <- log(unos_selected$can_last_serum_creat + 1)

which(colnames(unos_selected) == "can_last_serum_creat")

colnames(unos_selected)[88] <- "can_last_logserum_creat"

#####################################################################################

sum(is.na(unos_selected)) / (nrow(unos_selected)*ncol(unos_selected)) # sum of NA in the full data set is 2.36%

colSums(is.na(unos_selected)) # missing values per variable

colSums(is.na(unos_selected))[colSums(is.na(unos_selected)) > 10000 ] # all categorical

colSums(is.na(unos_selected))[colSums(is.na(unos_selected)) > 1000 & colSums(is.na(unos_selected)) < 10000] # 15 variables with more than 10000 NA

colSums(is.na(unos_selected))[colSums(is.na(unos_selected)) > 0 & colSums(is.na(unos_selected)) < 1000]

# show which variables have missing values out of the 101

vars_mis <- colnames(unos_selected)[sapply(colnames(unos_selected), function(x) sum(is.na(unos_selected[, x]))) > 0]

# cumulative death hazard is Ho(t), survival time T

library(mice)

# for overall survival

H0_t <- nelsonaalen(data = unos_selected,

timevar = patientsurvival,

statusvar = death)

cor(cbind(H0_t, Survival = unos_selected$patientsurvival,

Log = log(unos_selected$patientsurvival + 1))) # 0.989 correlation

# for failure-free survival

H0_t2 <- nelsonaalen(data = unos_selected,

timevar = gs_ffs,

statusvar = gs_ffsstate)

cor(cbind(H0_t2, Survival = unos_selected$gs_ffs,

Log = log(unos_selected$gs_ffs + 1))) # 0.988 correlation

# correlation almost 1, so for these data it matters little whether we take

# Ho(t) or T as a predictor

# using unsupervised splitting, original missForest algorithm

set.seed(12345)

time_rf_imput <- system.time(

unos_complete <- impute.rfsrc(data = unos_selected, ntree = 500, mf.q = 1,

max.iter = 5, do.trace = TRUE)

)

# round the values of some variables that were integers initially

unos_complete$donorage <- round(unos_complete$donorage, digits = 0)

unos_complete$recipientage <- round(unos_complete$recipientage, digits = 0)

save(unos_complete, file = "unos_complete.RData")

print(time_rf_imput / 3600) # elapsed time

# We grow a forest to impute the data. To proceed split statistics are calculated,

# If a node splits on a variable with

# missing data, the variable's missing data is imputed by randomly drawing values from nonmissing

# in-bag data. The purpose of this is to make it possible to assign cases to daughter

# nodes based on the split.

# splitting is implemented More precisely, mtry variables are selected at random,

# and for each of these a random subset of ytry variables are selected and defined as the

# multivariate pseudo-responses. A multivariate composite splitting rule of dimension ytry is

# then applied to each of the mtry multivariate regression problems and the node split on the

# variable leading to the best split

# We used all possible variable combinations as responses, and split by the rest of the variables using

# multivariate composite splitting.

# Missing data for responses are imputed by prediction. The process is repeated using a new set

# of variables for responses (mutually exclusive to the previous fit), until all variables have been

# imputed. The procedure is repeated until convergence of the algorithm. We set maximum number of iterations to 5.

# This is the most accurate of all imputation procedures that the randomForestSCR offers, but also by far the most

# computationally expensive one.

# apply administrative censoring at 10 years to the times and the statuses

unos_complete$death <- unos_complete$death*(unos_complete$patientsurvival <= 10)

unos_complete$patientsurvival <- pmin(unos_complete$patientsurvival, 10)

unos_complete$gs_ffsstate <- unos_complete$gs_ffsstate*(unos_complete$gs_ffs <= 10)

unos_complete$gs_ffs <- pmin(unos_complete$gs_ffs, 10)

sink("imputation_results.txt")

sum(is.na(unos_complete))

colnames(unos_complete)

unos_overall <- unos_complete[, 1:99]

unos_failure_free <- unos_complete[, c(1:97, 100, 101)]

# add one day to the survival time to avoid zero that can cause

# algorithm convergence problems

unos_overall$patientsurvival <- unos_overall$patientsurvival + (1/365.25)

unos_failure_free$gs_ffs <- unos_failure_free$gs_ffs + (1/365.25)

# let's see what values were imputed

# Categorical variables with more than 10000 NA

table(unos_complete$portal_hyperten_bleed[which(is.na(unos_selected$portal_hyperten_bleed))]) # imputed only N

table(unos_complete$rec_tumor[which(is.na(unos_selected$rec_tumor))]) # imputed only N

table(unos_complete$can_drug_treat_hyperten[which(is.na(unos_selected$can_drug_treat_hyperten))]) # imputed 558 Y

table(unos_complete$rec_bacteria_perit[which(is.na(unos_selected$rec_bacteria_perit))]) # imputed only N

table(unos_complete$can_variceal_bleeding[which(is.na(unos_selected$can_variceal_bleeding))]) # imputed only N

table(unos_complete$can_peptic_ulcer[which(is.na(unos_selected$can_peptic_ulcer))]) # imputed only No

table(unos_complete$can_angina_cad[which(is.na(unos_selected$can_angina_cad))]) # imputed only No

table(unos_complete$can_cereb_vasc[which(is.na(unos_selected$can_cereb_vasc))]) # imputed only N

table(unos_complete$can_periph_vasc[which(is.na(unos_selected$can_periph_vasc))]) # imputed only N

# Categorical with 1000 to 6000 NA

table(unos_complete$don_anti_convuls[which(is.na(unos_selected$don_anti_convuls))]) # imputed only N

table(unos_complete$malig[which(is.na(unos_selected$malig))]) # imputed only N

table(unos_complete$don_prev_gastro_disease[which(is.na(unos_selected$don_prev_gastro_disease))]) # imputed only N

# Let's see the summary statistics for some continuous variables before and afterwards

summary(unos_selected$don_loginr)

summary(unos_complete$don_loginr) # very similar results before and after

summary(unos_selected$rec_bmi)

summary(unos_complete$rec_bmi) # very similar results before and after

summary(unos_selected$rec_hgt_cm)

summary(unos_complete$rec_hgt_cm) # almost identical results before and after

sink()

# save the datasets

save(unos_overall, file = "unos_overall.RData")

save(unos_failure_free, file = "unos_failure_free.RData")

###############################################################################

# possible diagnostics after imputations for continuous variables

install_packages <- c("ggplot2", "gridExtra")

for (i in 1:length (install_packages)){

if (!install_packages[i] %in% installed.packages()){

install.packages(install_packages[i])

}

}

library(ggplot2)

library(gridExtra)

mat1 <- cbind(unos_selected$don_loginr[!is.na(unos_selected$don_loginr)],

"observed")

mat2 <- cbind(unos_complete$don_loginr[which(is.na(unos_selected$don_loginr))],

"imputed")

df <- as.data.frame(rbind(mat1, mat2))

df$V1 <- as.numeric(as.character(df$V1))

df$V2 <- as.factor(df$V2)

colnames(df) <- c("don_loginr", "Data")

plot1 <- ggplot(df, aes(don_loginr, colour = Data)) +

geom_density() +

xlab("Donor's log-transformed INR") +

xlim(c(-2, 2)) +

ggtitle(" ")

mat3 <- cbind(unos_selected$rec_bmi[!is.na(unos_selected$rec_bmi)],

"observed")

mat4 <- cbind(unos_complete$rec_bmi[which(is.na(unos_selected$rec_bmi))],

"imputed")

df2 <- as.data.frame(rbind(mat3, mat4))

df2$V1 <- as.numeric(as.character(df2$V1))

df2$V2 <- as.factor(df2$V2)

colnames(df2) <- c("rec_bmi", "Data")

plot2 <- ggplot(df2, aes(rec_bmi, colour = Data)) +

geom_density() +

xlab("BMI of the recipient") +

xlim(c(0, 50)) +

ggtitle(" ")

gridExtra::grid.arrange(plot1, plot2, ncol = 2)

# create training and test data

# load("unos_overall.RData") # contains 62294 observations and 99 variables

# load("unos_failure_free.RData") # contains 62294 observations and 99 variables

# first for unos_overall

N <- nrow(unos_overall)

round(table(unos_overall$death)/ N, 3) # event for 28.5% of the people

set.seed(1234)

index <- createDataPartition(unos_overall$death, p = 2/3, list = FALSE)

training_overall <- unos_overall[index, ]

test_overall <- unos_overall[-index,]

round(table(training_overall$death) / nrow(training_overall), 3)

round(table(test_overall$death) / nrow(test_overall), 3)

str(training_overall)

# save the partitioned data sets

save(training_overall, file = "training_overall.RData")

save(test_overall, file = "test_overall.RData")

# secondly for unos_failure_free

N <- nrow(unos_failure_free)

round(table(unos_failure_free$gs_ffsstate)/ N, 3)

set.seed(1234)

index <- createDataPartition(unos_failure_free$gs_ffsstate, p = 2/3, list = FALSE)

training_ffs <- unos_failure_free[index, ]

test_ffs <- unos_failure_free[-index,]

round(table(training_ffs$gs_ffsstate) / nrow(training_ffs), 3)

round(table(test_ffs$gs_ffsstate) / nrow(test_ffs), 3)

# save the partitioned data sets for ffs

save(training_ffs, file = "training_ffs.RData")

save(test_ffs, file = "test_ffs.RData")

####################################################################

# time distributions

####################################################################

load("training_ffs.RData")

install_packages <- c("ggplot2", "gridExtra")

for (i in 1:length (install_packages)){

if (!install_packages[i] %in% installed.packages()){

install.packages(install_packages[i])

}

}

library(ggplot2)

library(gridExtra)

plota <- ggplot(data = training_ffs, aes(gs_ffs)) +

geom_histogram(col="black", # purple

fill="darkgray",

alpha = .7,

bins = 12) +

labs(x = "Time since transplantation in years", y = "Frequency",

title = "Survival times for FFS") +

scale_x_continuous(breaks = seq(0, 12, by = 2)) +

scale_y_continuous(limits = c(0, 7000)) +

theme_bw() + #theme_grey

theme(plot.title = element_text(hjust = 0.5))

plotb <- ggplot(data = NULL,

aes(training_ffs$gs_ffs[training_ffs$gs_ffsstate == 1])) +

geom_histogram(col="black", # red

fill="gray85",

alpha = .7,

bins = 12) +

labs(x = "Time since transplantation in years", y = "Frequency",

title = "Event times for FFS") +

scale_x_continuous(breaks = seq(0, 12, by = 2)) +

scale_y_continuous(limits = c(0, 7000)) +

theme_bw() +

theme(plot.title = element_text(hjust = 0.5))

grid.arrange(plota, plotb, nrow = 1, ncol = 2)

####################################################################

# supplementary figure

####################################################################

install_packages <- c("survival", "dynpred")

for (i in 1:length (install_packages)){

if (!install_packages[i] %in% installed.packages()){

install.packages(install_packages[i])

}

}

# Figure in appendix: Survival and censoring functions for Data

library(survival)

library(dynpred)

load("training_ffs.RData")

unos_ffs_km <- survfit(formula = Surv(gs_ffs, gs_ffsstate) ~ 1, data = training_ffs) # 13034 events

unos_ffs_cens <- survfit(formula = Surv(gs_ffs, gs_ffsstate==0) ~ 1, data = training_ffs)

oldpar <- par(no.readonly=TRUE) # save graphical parameters

layout(matrix(1:2, 1, 2),widths=c(10.25,9))

par(mar= c(5, 4, 4, 0.1) + 0.1)

plot(unos_ffs_km, mark.time = FALSE, conf.int = FALSE, lwd=2, xlim = c(0, 10),

xlab = "Time (years since transplantation)", ylab = "Probability")

title(main="Survival")

par(mar = c(5, 0.1, 4, 1) + 0.1)

plot(unos_ffs_cens, mark.time = FALSE, conf.int = FALSE, lwd = 2, xlim = c(0, 10),

xlab = "Time (years since transplantation)", ylab = "", axes = FALSE)

axis(1)

box()

title(main="Censoring")

par(oldpar) # reset graphical parameters

####################################################################

# R code of the file: the functions2

####################################################################

install_packages <- c("survival", "pec",

"caret", "e1071")

for (i in 1:length (install_packages)){

if (!install_packages[i] %in% installed.packages()){

install.packages(install_packages[i])

}

}

# libraries to call

library(survival)

library(pec)

library(caret)

library(e1071)

# function that gets the probability matrix and returns the relative metrics

# for failure-free survival using the SORT-UNIQUE time points of test data

metrics_ffs_su <- function(prob_mat_su, test_set, p = 0.5) {

relative_probs <- vector(mode = "numeric", length = nrow(test_set))

time <- sort(unique(test_set$gs_ffs))

for (i in 1:length(relative_probs)){

temp <- prob_mat_su[i, ]

ind <- max(which(time == test_set$gs_ffs[i]))

relative_probs[i] <- temp[ind] # find the relative survival probability of the real survival interval

}

# find expected status based on 0.5 probability cut-off

expected_status <- ifelse(relative_probs > p, 0, 1)

classes <- c(0, 1)

tabel <- table(factor(test_set$gs_ffsstate, levels = classes),

factor(expected_status, levels = classes)) # auxiliary confusion table

confusion_mat <- confusionMatrix(tabel, positive = "1")

accuracy <- round(as.numeric(confusion_mat$overall[1]), 3)

sensitivity <- round(as.numeric(confusion_mat$byClass[1]), 3)

specificity <- round(as.numeric(confusion_mat$byClass[2]), 3)

precision <- round(as.numeric(confusion_mat$byClass[5]), 3)

recall <- round(as.numeric(confusion_mat$byClass[6]), 3)

f1score <- round(as.numeric(confusion_mat$byClass[7]), 3)

return(list(Accuracy = accuracy, Sensitivity = sensitivity,

Specificity = specificity, Precision = precision,

Recall = recall, F1score = f1score))

}

# EXAMPLE

# cox_all <- coxph(Surv(gs_ffs, gs_ffsstate) ~ ., data = training_ffs,

# method = "breslow", x = TRUE, y = TRUE)

# probs_cox_all_su <- predictSurvProb(object = cox_all, newdata = test_ffs,

# times = sort(unique(test_ffs$gs_ffs)))

#

# metrics_ffs_su(prob_mat_su = probs_cox_all_su, test_set = test_ffs)

# An adjusted function for the metrics for ffs that provides the metrics at "times"

metrics_ffs_new <- function(prob_mat, test_set, p = 0.5, times) {

relative_probs <- vector(mode = "numeric", length = nrow(test_set))

time <- times

for (i in 1:length(relative_probs)){

temp <- prob_mat[i, ]

ind <- max(which(time <= test_set$gs_ffs[i]))

relative_probs[i] <- temp[ind] # find the relative survival probability of the real survival interval

}

# find expected status based on 0.5 probability cut-off

expected_status <- ifelse(relative_probs > p, 0, 1)

classes <- c(0, 1)

tabel <- table(factor(test_set$gs_ffsstate, levels = classes),

factor(expected_status, levels = classes)) # auxiliary confusion table

confusion_mat <- confusionMatrix(tabel, positive = "1")

accuracy <- round(as.numeric(confusion_mat$overall[1]), 3)

sensitivity <- round(as.numeric(confusion_mat$byClass[1]), 3)

specificity <- round(as.numeric(confusion_mat$byClass[2]), 3)

precision <- round(as.numeric(confusion_mat$byClass[5]), 3)

recall <- round(as.numeric(confusion_mat$byClass[6]), 3)

f1score <- round(as.numeric(confusion_mat$byClass[7]), 3)

return(list(Accuracy = accuracy, Sensitivity = sensitivity,

Specificity = specificity, Precision = precision,

Recall = recall, F1score = f1score))

}

# EXAMPLE

# probs_cox_all <- predictSurvProb(object = cox_all, newdata = test_ffs,

# times = seq(0, 10, length.out = 101))

# metrics_ffs_new(prob_mat_su = probs_cox_all, test_set = test_ffs,

# times = seq(0, 10, length.out = 101))

# Estimate the Brier score at the sort-unique time points

brier_general_su <- function(prob_matrix, data) {

f_mod <- Surv(gs_ffs, gs_ffsstate) ~ 1

PredError <- pec(object = prob_matrix,

formula = f_mod,

cens.model = "marginal",

data = data,

times = sort(unique(data$gs_ffs)),

exact = FALSE,

splitMethod="none")

timing <- 0:(length(PredError$AppErr$matrix) - 1)

indexa <- 2:length(timing)

int_bs <- (diff(timing) %*% ((PredError$AppErr$matrix[indexa - 1]

+ PredError$AppErr$matrix[indexa])/2))/diff(range(timing))

return(list(Time = c(0, sort(unique(test_ffs$gs_ffs))),

Brier = round(PredError$AppErr$matrix, 3),

Int_brier = round(int_bs, 3)))

}

# EXAMPLE

# cox_all <- coxph(Surv(gs_ffs, gs_ffsstate) ~ ., data = training_ffs,

# method = "breslow", x = TRUE, y = TRUE)

# probs_cox_all <- predictSurvProb(object = cox_all, newdata = test_ffs,

# times = c(0, sort(unique(test_ffs$gs_ffs))))

# brier_general_su(prob_matrix = probs_cox_all, data = test_ffs)

# Estimate Brier score at specific time point

brier_general_new <- function(prob_matrix, data, times) {

f_mod <- Surv(gs_ffs, gs_ffsstate) ~ 1

PredError <- pec(object = prob_matrix,

formula = f_mod,

cens.model = "marginal",

data = data,

times = times,

exact = FALSE,

splitMethod="none")

timing <- 0:(length(PredError$AppErr$matrix) - 1)

indexa <- 2:length(timing)

int_bs <- (diff(timing) %*% ((PredError$AppErr$matrix[indexa - 1]

+ PredError$AppErr$matrix[indexa])/2))/diff(range(timing))

return(list(Time = times, Brier = round(PredError$AppErr$matrix, 3),

Int_brier = round(int_bs, 3)))

}

# EXAMPLE

# times <- seq(0, 10, length.out = 101)

# probs_cox_all <- predictSurvProb(object = cox_all, newdata = test_ffs,

# times = times)

# brier_general_new(prob_matrix = probs_cox_all, data = test_ffs,

# times = times)

# calculate Brier and Integrated Brier scores for the neural networks

# prob matrix is of the form nrow(data) x 11

brier_nnet <- function(prob_matrix, data) {

f_nnet <- Surv(gs_ffs, gs_ffsstate) ~ 1

# we calculate the Brier score utilizing the pec package

# we subtract 1 day at 10 years to avoid zero censoring prob weight at this time

bsc <- pec(object = prob_matrix,

formula = f_nnet, cens.model = "marginal",

data = data, times = c(0, 1, 2, 3, 4, 5, 6, 7, 8, 9, 9.9973),

exact = FALSE, splitMethod="none")

# calculate integrated brier score

timing <- 0:(length(bsc$AppErr$matrix) - 1)

indexa <- 2:length(timing)

int_bs <- (diff(timing) %*% ((bsc$AppErr$matrix[indexa - 1]

+ bsc$AppErr$matrix[indexa])/2))/diff(range(timing))

return(list(Time = seq(0, 10, length.out = 11),

Brier = round(bsc$AppErr$matrix, 3),

Int_brier = round(int_bs, 3)))

}

########################################################################################################

# this is connection weight method performed in keras

# it extracts the variable importance from a NN with 1 hidden layer

var_imp_keras <- function(model){

list_weights <- get_weights(model)

# list_weights[[1]] are the weights between input-hidden node

# list_weights[[2]] are the weights between bias-hidden node

# list_weights[[3]] are the weights between hidden node-output

# list_weights[[4]] is the weight between bias-output

# names input nodes , number input nodes

# extract the column names from created data training_ffs_long: the covariates 1:119

# and additional the 10 time variables times.1 to times.10

names <- c(colnames(training_ffs_long)[1:119], colnames(training_ffs_long)[126:135])

# n_input <- length(list_weights[[1]]) / length(list_weights[[2]])

# number hidden nodes , number output nodes

# n_nodes <- length(list_weights[[3]])

# n_outputs <- length(list_weights[[4]])

# matrix multiplication input x hidden with hidden x output

mega_mat <- t(list_weights[[1]] %*% list_weights[[3]])

colnames(mega_mat) <- names

mega_mat_abs <- abs(mega_mat)

totals <- sum(mega_mat_abs)

mega_mat_rel <- as.data.frame(mega_mat_abs/ totals)

rels <- as.vector(as.numeric(mega_mat_rel))

return(list(Names = names, Rels = round(rels, 3)))

}

var_imp_keras2h <- function(model){

list_weights <- get_weights(model)

# list_weights[[1]] are the weights between input-hidden1

# list_weights[[2]] are the weights bias input

# list_weights[[3]] are the weights between hidden1-hidden2

# list_weights[[4]] are the weights bias hidden1

# list_weights[[5]] are the weights between hidden2-output

# list_weights[[2]] are the weights bias hidden2

# names input nodes , number input nodes

# extract the column names from created data training_ffs_long: the covariates 1:119

# and additional the 10 time variables times.1 to times.10

names <- c(colnames(training_ffs_long)[1:119], colnames(training_ffs_long)[126:135])

# n_input <- length(list_weights[[1]]) / length(list_weights[[2]])

# number hidden nodes , number output nodes

# n_nodes <- 2*length(list_weights[[3]]) # because you have 2 hidden layers with same nodes

# n_outputs <- length(list_weights[[4]])

# matrix multiplication input x hidden with hidden x output

mega_mat <- t(list_weights[[1]] %*% list_weights[[3]] %*% list_weights[[5]])

colnames(mega_mat) <- names

mega_mat_abs <- abs(mega_mat)

totals <- sum(mega_mat_abs)

mega_mat_rel <- as.data.frame(mega_mat_abs/ totals)

rels <- as.vector(as.numeric(mega_mat_rel))

return(list(Names = names, Rels = round(rels, 3)))

}

####################################################################

# Cox model with all variables and Cox backward

####################################################################

install_packages <- c("survival", "survminer", "rms", "ggplot2",

"grid", "gridExtra", "Hmisc", "MASS",

"survAUC", "pec", "dynpred")

for (i in 1:length(install_packages)){

if (!install_packages[i] %in% installed.packages()){

install.packages(install_packages[i])

}

}

library(survival)

library(survminer)

library(rms)

library(ggplot2)

library(gridExtra)

library(pec)

library(dynpred)

# source("https://bioconductor.org/biocLite.R")

# biocLite("survcomp")

library(survcomp)

load("training_ffs.RData")

load("test_ffs.RData")

source("the_functions2.R")

# new coding for master thesis article

cox_all <- coxph(Surv(gs_ffs, gs_ffsstate) ~ ., data = training_ffs,

method = "breslow", x = TRUE, y = TRUE)

res <- summary(cox_all)

f_cox_all <- as.formula(paste("Surv(gs_ffs, gs_ffsstate)~",

paste(colnames(training_ffs)[1:97], collapse="+")))

test_ph <- cox.zph(cox_all)

round(test_ph$table[, 3], digits = 3)

# 17 variables (19 var levels) violate the proportionality of hazards assumption

round(test_ph$table[, 3], digits = 3)[which(test_ph$table[, 3] < 0.05)]

# coefficients from the Cox model

res_nolist <- unlist(res$coefficients)

# coefficients from the Cox model sorted by Z-score value

round(res_nolist[order(res_nolist[, 4], decreasing = T), ][1:12, ], digits = 3)

vars <- c("retransplantationRetransplantation", "donorage",

"donortypeDCD", "log_coldischemiatime", "diabY", "can_race_srtrBlack",

"lifesupportLifesupport", "recipientage", "rec_tumorY",

"portal_hyperten_bleedY", "hcvY", "pretxstatusIC_UNIT")

ind <- rownames(res$conf.int) %in% vars

round(res$conf.int[ind, ], 3)

# first we run some univariate analysis to get some insight about the variables

variable_list <- colnames(training_ffs)[1:97]

p_vals <- vector(mode = "numeric", length = length(variable_list))

univariate_testing <- function(var, data) {

obj <- summary(coxph(Surv(gs_ffs, gs_ffsstate) ~ data[, var],

data = data, method = "breslow"))

return(round(as.numeric(obj$waldtest["pvalue"]), digits = 4))

}

for (i in 1:length(variable_list)) {

p_vals[i] <- univariate_testing(variable_list[i], data = training_ffs)

}

# univariate testing Wald p-value

df <- data.frame(Variable = variable_list,

P_value = as.numeric(p_vals))

capture.output(print(df), file = "univariate_analysis_ffs.txt")

sig_df <- df[df$P_value < 0.05, ] # 71 prognostic in univariate analyses

rownames(sig_df) <- NULL

sig_df

# Predictions for the test data

pred_cox_all <- predict(cox_all,

newdata = test_ffs[, -c(98, 99)], type = "lp")

conc_cox_all <- concordance.index(pred_cox_all,

surv.time = test_ffs$gs_ffs,

surv.event = test_ffs$gs_ffsstate)

conc_cox_all$c.index

conc_cox_all$lower

conc_cox_all$upper

# Brier score using pec

f_cox_all <- as.formula(paste("Surv(gs_ffs, gs_ffsstate)~",

paste(colnames(test_ffs)[1:97], collapse="+")))

PredError <- pec(object = cox_all,

formula = f_cox_all, cens.model = "marginal",

data = test_ffs, start = 0, maxtime = 10,

exactness = 100, exact = FALSE, splitMethod="none")

plot(PredError)

mean(PredError$AppErr$coxph)

# integrated Brier score

timing <- PredError$time

indexa <- 2:length(timing)

int_bs <- (diff(timing) %*% ((PredError$AppErr$coxph[indexa - 1] + PredError$AppErr$coxph[indexa])/2))/diff(range(timing))

# 2nd way providing the probability matrix

# dim(probs_all)

probs_cox_all <- predictSurvProb(object = cox_all, newdata = test_ffs,

times = seq(0, 10, length.out = 101)) # to have exactness 100

PredError <- pec(object = probs_cox_all, formula = f_cox_all,

cens.model = "marginal",

data = test_ffs, start = 0, maxtime = 10,

exactness = 100, exact = FALSE, splitMethod="none")

plot(PredError)

mean(PredError$AppErr$matrix)

# calculate with the handmade function at times

times <- seq(0, 10, length.out = 101)

PredError <- brier_general_new(prob_matrix = probs_cox_all,

data = test_ffs, times = times)

mean(PredError$Brier)

plot(x = PredError$Time, y = PredError$Brier,

type = "l", xlab = "Time in years since transplantation",

ylab = "Brier score", ylim = c(0, 0.33),

main = "Brier score of the Cox model with all variables")

# Brier score with my function

times <- seq(0, 10, length.out = 11)

probs_cox_all <- predictSurvProb(object = cox_all, newdata = test_ffs,

times = times)

colMeans(probs_cox_all)

PredError <- brier_general_new(prob_matrix = probs_cox_all,

data = test_ffs, times = times)

PredError$Brier

PredError$Int_brier

plot(x = PredError$Time, y = PredError$Brier,

type = "l", xlab = "Time in years since transplantation",

ylab = "Brier score", ylim = c(0, 0.33),

main = "Brier score of the Cox model with all variables")

# Cox backward

cox_back <- selectCox(formula = Surv(gs_ffs, gs_ffsstate) ~.,

data = training_ffs, rule = "aic") # 34 variables in total

cox_back$fit

# backward elimination using the rms R package of Frank Harrell selected 34 variables

vars_back <- cox_back$In # variables

f_back <- as.formula(paste("Surv(gs_ffs, gs_ffsstate) ~",

paste(vars_back, collapse = "+")))

cox_back <- coxph(formula = f_back, x = TRUE, y = TRUE,

data = training_ffs, method = "breslow")

sum2 <- summary(cox_back)

ind2 <- rownames(sum2$conf.int) %in% vars

round(sum2$conf.int[ind2, ], 3)

anova(cox_all, cox_back)

# the higher the value of log-lik the better

logLik(cox_all); logLik(cox_back)

# the lower the AIC/BIC the better

AIC(cox_all); AIC(cox_back)

BIC(cox_all); BIC(cox_back)

# diagnostics for Cox backward

temp <- cox.zph(cox_back)

indx <- round(temp$table, 3)[, 3] < 0.05

round(temp$table, 3)[, 3][indx] # Cox PH violated for 16 variables

# predictions on the test data

pred_test_back <- predict(cox_back,

newdata = test_ffs[, -c(98, 99)], type = "lp")

conc_back <- concordance.index(pred_test_back,

surv.time = test_ffs$gs_ffs,

surv.event = test_ffs$gs_ffsstate)

conc_back$c.index

conc_back$lower

conc_back$upper

# Brier score with pec

probs_cox_back <- predictSurvProb(object = cox_back, newdata = test_ffs,

times = seq(0, 10, length.out = 101)) # to have exactness 100

PredError <- pec(object = probs_cox_back, formula = Surv(gs_ffs, gs_ffsstate) ~ 1,

cens.model = "marginal",

data = test_ffs, start = 0, maxtime = 10,

exactness = 100, exact = FALSE, splitMethod="none")

plot(PredError)

# Brier score with my function

times <- seq(0, 10, length.out = 11)

probs_cox_back <- predictSurvProb(object = cox_back, newdata = test_ffs,

times = times)

colMeans(probs_cox_back)

PredError <- brier_general_new(prob_matrix = probs_cox_back,

data = test_ffs, times = times)

PredError$Brier

PredError$Int_brier

plot(x = PredError$Time, y = PredError$Brier,

type = "l", xlab = "Time in years since transplantation",

ylab = "Brier score", ylim = c(0, 0.33),

main = "Brier score of the Cox model with backward selection")

# Calibration plots

f_full <- Surv(gs_ffs, gs_ffsstate) ~ .

calPlot(cox_all, formula = f_full,

time = 6, data = test_ffs, bars = TRUE)

# calibration plot

calPlot(cox_back, formula = f_back,

time = 6, data = test_ffs, bars = TRUE)

####################################################################

# Cox model with LASSO

####################################################################

install_packages <- c("survival", "glmnet",

"hdnom", "caret")

for (i in 1:length (install_packages)){

if (!install_packages[i] %in% installed.packages()){

install.packages(install_packages[i])

}

}

library(survival)

library(glmnet)

library(survcomp)

library(hdnom)

library(caret)

source("the_functions2.R")

load("training_ffs.RData")

load("test_ffs.RData")

response_pair <- c("gs_ffs", "gs_ffsstate")

# for the training set

X_train <- training_ffs[, !(colnames(training_ffs) %in% response_pair)]

X_train_ffs <- model.matrix(~., X_train)[, -1] # create martix X for glmnet

# dim(X_train_ffs) # 41530 x 119

Y_train_ffs <- training_ffs[, colnames(training_ffs) %in% response_pair] # create matrix Y

Y_survtrain_ffs <- Surv(Y_train_ffs$gs_ffs, Y_train_ffs$gs_ffsstate)

# for the test set

x_test <- test_ffs[, !(colnames(test_ffs) %in% response_pair)]

X_test_ffs <- model.matrix(~., x_test)[, -1]

Y_test_ffs <- test_ffs[, colnames(test_ffs) %in% response_pair]

# Surv function packages survival data into the form expected by glmnet

Y_survtest_ffs <- Surv(Y_test_ffs$gs_ffs, Y_test_ffs$gs_ffsstate)

# use these two datasets to fit Cox model in the selected features

training_ffs_extended <- as.data.frame(cbind(X_train_ffs, Y_train_ffs))

test_ffs_extended <- as.data.frame(cbind(X_test_ffs, Y_test_ffs))

# save(training_ffs_extended, file = "training_ffs_extended.RData")

# save(test_ffs_extended, file = "test_ffs_extended.RData")

library(doParallel)

if (!exists("cl")) {

cl <- makeCluster(1)

registerDoParallel(cl)

}

# getDoParWorkers()

# getDoParName()

# stopCluster(cl)

set.seed(1234)

folds <- createFolds(y = training_ffs$gs_ffs, k = 5, list = FALSE)

time_cv <- system.time(

cv.fit <- cv.glmnet(x = X_train_ffs, y = Y_survtrain_ffs,

foldid = folds, parallel = TRUE,

family = "cox", grouped = TRUE,

maxit = 1000)

)

cv.fit$glmnet.fit

cv.fit$lambda.min # value of lambda that gives minimum cvm

# plot(cv.fit$glmnet.fit)

# plot(cv.fit$lambda, cv.fit$cvm)

plot(cv.fit) # 40 variables are selected with the ad-hoc 1se rule

# left vertical line: where cv error curve hits the minimum

# right vertical line: the most regularized model with cv-error within 1-sd of minimum

plot(cv.fit, sign.lambda = -1)

# Fit a generalized linear model via penalized maximum likelihood, alpha = 1 means Lasso

fit <- glmnet(x = X_train_ffs, y = Y_survtrain_ffs, alpha = 1,

lambda = cv.fit$lambda.1se,

family = "cox", maxit = 1000)

# returns the p length coef vector of the solution

coefficients <- coef(fit, s = cv.fit$lambda.1se)

# available coefficients for test set, the same will be non-zero as the train set

names_coefs <- unlist(coefficients@Dimnames[1])

# corresponding to lambda = cv.fit$lambda.min.

active_index <- which(coefficients != 0)

active_coefs <- coefficients[active_index]

vars_active_1se <- names_coefs[active_index]

df <- data.frame(Variable = vars_active_1se,

Coef = round(active_coefs, 3),

Exp_Coef = round(exp(active_coefs), 3))

rownames(df) <- NULL

vars <- c("retransplantationRetransplantation", "donorage",

"donortypeDCD", "log_coldischemiatime", "diabY", "can_race_srtrBlack",

"lifesupportLifesupport", "recipientage", "rec_tumorY",

"portal_hyperten_bleedY", "hcvY", "pretxstatusIC_UNIT")

# check the results for the 10 most prognostic variables for the Cox with all variables

indx <- df$Variable %in% vars

df[indx, ]

# predictions for the Cox LASSO model

response_pair <- data.frame(time = test_ffs$gs_ffs, status = test_ffs$gs_ffsstate)

surv_pred <- predict(cv.fit, newx = X_test_ffs,

s = "lambda.1se", type="link") # gives linear predictors - same as lp

hist(surv_pred) # non-centered linear predictors

concordance.index(x = surv_pred, surv.time = test_ffs_extended$gs_ffs,

surv.event = test_ffs_extended$gs_ffsstate)$c.index # 0.6192

# function to estimate survival probabilities from a glmnet object

predictProb_glmnet <- function (glmnet_object, response_pair, x, times)

{

lp <- as.numeric(predict(glmnet_object, newx = data.matrix(x),

s = "lambda.1se", type="link"))

basesurv <- glmnet_basesurv(time = response_pair$time,

event = response_pair$status,

lp = lp, times.eval = times)

p <- exp(exp(lp) %*% -t(basesurv$cumulative_base_hazard))

if (NROW(p) != NROW(x) || NCOL(p) != length(times))

stop("Prediction failed")

return(p)

}

probs_lasso <- predictProb_glmnet(glmnet_object = cv.fit,

response_pair = response_pair,

x = X_test_ffs,

times = seq(0, 10, length.out = 101))

colMeans(probs_lasso)

# calculating the Brier score with pec

f_lasso <- Surv(gs_ffs, gs_ffsstate) ~ 1

PredError <- pec(object = probs_lasso, formula = f_lasso, cens.model = "marginal",

data = test_ffs_extended, start = 0, maxtime = 10,

exactness = 100, exact = FALSE, splitMethod="none")

plot(PredError)

mean(PredError$AppErr$matrix)

# Brier score with my function

probs_lasso <- predictProb_glmnet(glmnet_object = cv.fit,

response_pair = response_pair,

x = X_test_ffs,

times = seq(0, 10, length.out = 11))

PredError <- brier_general_new(prob_matrix = probs_lasso, data = test_ffs_extended,

times = seq(0, 10, length.out = 11))

as.numeric(PredError$Int_brier)

plot(x = PredError$Time, y = PredError$Brier,

type = "l", xlab = "Time in years since transplantation",

ylab = "Brier score", ylim = c(0, 0.33),

main = "Brier score of the Cox LASSO")

####################################################################

# Random Survival forest: quick tuning

####################################################################

install_packages <- c("survival", "randomForestSRC", "caret")

for (i in 1:length (install_packages)){

if (!install_packages[i] %in% installed.packages()){

install.packages(install_packages[i])

}

}

library(survival)

library(caret)

library(randomForestSRC)

library(parallel)

options(rf.cores = 20, mc.cores = 20)

load("training_ffs.RData")

load("test_ffs.RData")

cat("Starting forest tuning ... ")

forest_tuning1 <- tune.rfsrc(Surv(gs_ffs, gs_ffsstate) ~ .,

data = training_ffs, ntreeTry = 250,

#sampsize = 10000, ntime = 1000,

nodesizeTry = c(5, seq(10, 100, by = 10)),

trace = TRUE, maxIter = 20, doBest = TRUE)

forest_tuning1$optimal

cat("Starting forest tuning 2 ... ")

forest_tuning2 <- tune.rfsrc(Surv(gs_ffs, gs_ffsstate) ~ .,

data = training_ffs, ntreeTry = 250,

#sampsize = 10000, ntime = 1000,

nodesizeTry = c(5, seq(10, 100, by = 10)),

trace = TRUE, maxIter = 20, doBest = TRUE)

forest_tuning2$optimal

cat("Starting forest tuning 3 ... ")

forest_tuning3 <- tune.rfsrc(Surv(gs_ffs, gs_ffsstate) ~ .,

data = training_ffs, ntreeTry = 250,

#sampsize = 10000, ntime = 1000,

nodesizeTry = c(5, seq(10, 100, by = 10)),

trace = TRUE, maxIter = 20, doBest = TRUE)

forest_tuning3$optimal

optimals <- data.frame(combis1 = forest_tuning1$optimal,

combis2 = forest_tuning2$optimal,

combis3 = forest_tuning3$optimal)

save(optimals, file = "quicktune_optimals_ffs.RData")

####################################################################

# Random Survival forest: 5-fold cross-validation

####################################################################

install_packages <- c("survival", "randomForestSRC", "caret")

for (i in 1:length (install_packages)){

if (!install_packages[i] %in% installed.packages()){

install.packages(install_packages[i])

}

}

# cross validation random forest ffs

library(survival)

library(caret)

library(randomForestSRC)

library(parallel)

options(rf.cores = 60, mc.cores = 60)

load("training_ffs.RData")

load("test_ffs.RData")

###################################################################################################

# cross-validation to find best values of (ntree, splitrule) nsplit, nodesize, maybe mtry

# mtry: Number of variables randomly selected as candidates for splitting a node.

# nsplit: a maximum of nsplit split points are randomly chosen among possible split points of x var

# nodesize: forest average number of unique cases in terminal node

nodesize <- c(10, 20, 35, 50, 70, 85, 100)

nsplit <- c(3, 4, 5, 6, 7)

mtry <- c(5, 12, 19, 26, 33, 40, 47)

combis <- expand.grid(nodesize, nsplit, mtry)

nfolds <- 5

set.seed(12345)

folds <- createFolds(training_ffs$gs_ffs, k = 5, list = TRUE)

cv_time <- matrix(0, nrow = nfolds, ncol = nrow(combis))

oob_error <- matrix(0, nrow = nfolds, ncol = nrow(combis))

for (i in 1:nfolds) {

cat("Starting iteration i = ", i, "\n")

indices <- folds[[i]]

train_set <- training_ffs[-indices, ]

validation_set <- training_ffs[indices, ]

for (j in 1:nrow(combis)) {

cat("Testing combination number:", j, "of repeat", i, " out of 5", "\n")

cat("It is:", "nodesize: ", combis[j, 1],

"nsplit: ", combis[j, 2], "and mtry: ", combis[j, 3], "\n")

cv_time[i, j] <- {

system.time(

fit_ffs <- rfsrc(Surv(gs_ffs, gs_ffsstate) ~ ., splitrule = "logrank",

nodesize = combis[j, 1], nsplit = combis[j, 2],

data = train_set, mtry = combis[j, 3],

ntree = 500, seed = -12345, forest = TRUE))[3]

}

cat("Calculating the OOB validated prediction error ...", "\n")

fit_val <- predict(fit_ffs, newdata = validation_set,

seed = -12345, forest = FALSE)

oob_error[i, j] <- as.numeric(fit_val$err.rate[fit_val$ntree])

cat("Error is: ", oob_error[i, j], "\n")

}

}

df_logrank_ffs <- data.frame(Node_size = combis$Var1, Nsplit = combis$Var2,

Mtry = combis$Var3, Error = round(colMeans(oob_error), 4))

save(df_logrank_ffs, file = "df_logrank_ffs.Rdata")

save.image("image_logrank_ffs.Rdata")

####################################################################

# Random Survival forest: number of trees needed

####################################################################

install_packages <- c("survival", "randomForestSRC", "caret")

for (i in 1:length (install_packages)){

if (!install_packages[i] %in% installed.packages()){

install.packages(install_packages[i])

}

}

# Random forest ffs final code

library(survival)

library(caret)

library(randomForestSRC)

library(parallel)

options(rf.cores = 30, mc.cores = 30)

load("training_ffs.RData")

load("test_ffs.RData")

# fit the random survival forest with the tuned parameters from the cv

fit_block <- rfsrc(Surv(gs_ffs, gs_ffsstate) ~ ., splitrule = "logrank", nsplit = 5,

data = training_ffs, ntree = 500, split.depth = "all.trees",

var.used = "all.trees", seed = -12345, mtry = 12, nodesize = 50,

sampsize = nrow(training_ffs) / 2, block.size = 5, forest = FALSE

)

plot(fit_block)

jpeg("plot_trees_ffs.jpg")

plot(fit_block)

dev.off()

#plot_obj <- recordPlot()

#save.image("image_ntree_ffs.Rdata")

cat("Plot of number of trees recorded!")

####################################################################

# Random Survival forest: using the final model to estimate C-index

# the Brier and Integrated Brier Score as well as VIMP, minimal depth

####################################################################

install_packages <- c("survival", "randomForestSRC", "caret",

"prodlim", "pec")

for (i in 1:length (install_packages)){

if (!install_packages[i] %in% installed.packages()){

install.packages(install_packages[i])

}

}

# Random forest ffs final code

library(survival)

library(caret)

library(randomForestSRC)

library(parallel)

library(prodlim)

library(pec)

options(rf.cores = 70, mc.cores = 70)

load("training_ffs.RData")

load("test_ffs.RData")

cat("Fitting random forest... ")

# final fit ffs

fit <- rfsrc(Surv(gs_ffs, gs_ffsstate) ~ ., splitrule = "logrank", nsplit = 5,

data = training_ffs, ntree = 300, split.depth = "all.trees",

var.used = "all.trees", seed = -12345, mtry = 12, nodesize = 50,

sampsize = nrow(training_ffs) / 2, forest = TRUE, importance = TRUE

)

fit$err.rate[fit$ntree] # error on the training data

jpeg("plot_oob_mort.png", width = 600)

plot.survival.rfsrc(fit, plots.one.page = FALSE)

dev.off()

jpeg("plot_mortal_meds.png", width = 600)

plot.variable(fit, xvar.names = "retransplantation",

surv.type = "mort")

dev.off()

jpeg("plot_mortal_los.png", width = 600)

plot.variable(fit, xvar.names = "donorage",

surv.type = "mort")

dev.off()

leaf_count <- fit$leaf.count # number of terminal nodes for each tree in the forest

jpeg("plot_nr_leaves.png")

ggplot(NULL, aes(x = leaf_count)) +

geom_histogram(aes(y =..density..), bins = 20,

fill=I("lightblue"), color = I("blue")) +

geom_density(color = 2) +

labs(x = "Number of leaves per tree") +

theme(plot.title = element_text(hjust = 0.5))

dev.off()

vars_used <- tail(sort(fit$var.used), 10)

jpeg("plot_var_used.png", width = 750)

barplot(tail(sort(fit$var.used), 5), ylab = "Frequency", cex.names = 0.71)

dev.off()

# very important variables are used early

jpeg("plot_split_depth.png", width = 650)

hist(fit$split.depth, xlab = "Split depth per variable",

col = "red", main = "", breaks = 20)

dev.off()

save.image("image_rsf_ffs_final.RData")

# method for variable selection: minimal depth

vars1 <- var.select(object = fit, method = "md", conservative = "high",

refit = FALSE)

md <- vars1$varselect[1:20, ]

vars1$topvars

merged <- data.frame(Variable = rownames(md)[1:10], Depth = md$depth[1:10],

VIMP = md$vimp[1:10], Variable = rownames(md)[11:20],

Depth = md$depth[11:20], VIMP = md$vimp[11:20])

vars2 <- fit$importance

head(sort(vars2, decreasing = TRUE), 20)

a <- head(sort(vars2, decreasing = TRUE), 20)

df <- data.frame(Variable = attr(a, "names")[1:10], VIMP = round(a, 4)[1:10],

Variable = attr(a, "names")[11:20], VIMP = round(a, 4)[11:20])

rownames(df) <- NULL

df

cat("Starting variable importance on test set...")

#test data vimp

vars3 <- vimp(object = fit, newdata = test_ffs, importance = "permute")

print(vars3$importance)

b <- head(sort(vars3$importance, decreasing = TRUE), 20)

df2 <- data.frame(Variable = attr(b, "names")[1:10],

VIMP = round(b, 4)[1:10],

Variable = attr(b, "names")[11:20],

VIMP = round(b, 4)[11:20])

rownames(df2) <- NULL

df2

cat("Starting subsampling method for vimp ... ")

# plot subsample

fit_sub <- subsample(fit)

jpeg("plot_vimp_ci.png", width = 700)

plot.subsample(fit_sub, pmax = 8, cex = 0.55,

xlab = "100 x vimp (for survival time)")

dev.off()

save.image("image_rsf_ffs_final.RData")

###################################################################################################

# predictions on test set

fit_test <- predict(fit, newdata = test_ffs, seed = -12345,

split.depth = "all.trees", forest = FALSE)

cindex_rsf <- 1 - fit_test$err.rate[fit_test$ntree]

cat("The C-index on the test data is", cindex_rsf)

# always put the times in sorted order

probs_rsf <- predictSurvProb(object = fit, newdata = test_ffs,

times = seq(0, 10, length.out = 11))

save(probs_rsf, file = "probs_rsf.RData")

source("the_functions2.R")

# Brier score using pec

f_rsf <- as.formula("Surv(gs_ffs, gs_ffsstate) ~ 1")

PredError_rsf <- brier_general_new(prob_matrix = probs_rsf,

data = test_ffs,

times = seq(0, 10, length.out = 11))

plot(PredError_rsf)

PredError_rsf

save(PredError_rsf, file = "PredError_rsf.RData")

metrics_rsf_ffs <- metrics_ffs_new(probs_rsf, test_ffs,

times = seq(0, 10, length.out = 11))

save.image("image_rsf_ffs_final.RData")

metrics_rsf_ffs

# create the 3D plot of the supplementary material

# nice colorscales: "Viridis", "Electric", "Greys", "Reds"

library(plotly)

plot_ly(df_logrank_ffs, x = ~Node_size, y = ~Nsplit, z = ~Mtry,

marker = list(color = ~Error,

colorscale = "Viridis",

showscale = TRUE)) %>%

add_markers() %>%

layout(scene = list(xaxis = list(title = 'Nodesize'),

yaxis = list(title = 'Nsplit'),

zaxis = list(title = 'Mtry')),

annotations = list(

x = 1.00,

y = 1.05,

z = 0.90,

text = 'Prediction error',

xref = 'paper',

yref = 'paper',

showarrow = FALSE

))

####################################################################

# Neural networks: training example for 1 hidden layer

####################################################################

install_packages <- c("fastDummies", "keras", "survival", "pec",

"caret", "doParallel")

for (i in 1:length (install_packages)){

if (!install_packages[i] %in% installed.packages()){

install.packages(install_packages[i])

}

}

# Survival neural networks

library(fastDummies)

library(keras)

library(survival)

library(pec)

library(caret)

library(doParallel)

cl <- makeCluster(2)

registerDoParallel(cl)

# read the scaled training data for ffs (this is the scaled short format)

load("training_ffs_scaled.RData")

load("test_ffs_scaled.RData")

load("training_ffs_long.RData")

load("test_ffs_long.RData")

# training_ffs_long <- data_train_creator(data = training_ffs_scaled)

# validation_ffs_long <- data_test_creator(data = training_ffs_scaled)

# test_ffs_long <- data_test_creator(data = test_ffs_scaled)

# save(training_ffs_long, file = "training_ffs_long.RData")

# save(validation_ffs_long, file = "validation_ffs_long.RData")

# save(test_ffs_long, file = "test_ffs_long.RData")

# load file the functions

source("the_functions2.R")

#######################################################################################################

#######################################################################################################

# set up the cross-validation

# most popular optimization algorithms used are the Stochastic Gradient Descent (SGD), ADAM and RMSprop

# you need to tune certain parameters such as learning rate or momentum

nfolds <- 5

set.seed(1234)

folds <- createFolds(training_ffs_scaled$gs_ffs, k = 5, list = TRUE)

node_size <- seq(10, 130, by = 20) # grid of node sizes

dropout_rate <- c(0.1, 0.2, 0.3)

lr <- c(0.01, 0.1, 0.2)

class_weights <- c(1, 2.236) # increase the weight of weak class according to 69.1 / 30.9

momentum <- c(0.8, 0.9)

combis <- expand.grid(node_size, dropout_rate, lr, class_weights, momentum)

# initialize objects

# cv_error <- matrix(0, nrow = nfolds, ncol = nrow(combis))

cv_accuracy <- matrix(0, nrow = nfolds, ncol = nrow(combis))

cv_specificity <- matrix(0, nrow = nfolds, ncol = nrow(combis))

cv_sensitivity <- matrix(0, nrow = nfolds, ncol = nrow(combis))

cv_precision <- matrix(0, nrow = nfolds, ncol = nrow(combis))

cv_recall <- matrix(0, nrow = nfolds, ncol = nrow(combis))

cv_f1score <- matrix(0, nrow = nfolds, ncol = nrow(combis))

cv_weights <- matrix(0, nrow = nfolds, ncol = nrow(combis))

cv_intbrier <- matrix(0, nrow = nfolds, ncol = nrow(combis))

cv_relimp <- list(vector(mode = "list", length = nrow(combis)),

vector(mode = "list", length = nrow(combis)),

vector(mode = "list", length = nrow(combis)),

vector(mode = "list", length = nrow(combis)),

vector(mode = "list", length = nrow(combis)))

data_train_creator <- function(data){

N <- nrow(data)

# assign survival times to 10 intervals, each is a 1-year period

data$interval <- cut(data$gs_ffs, breaks = 10, labels = FALSE)

data$survival <- cut(data$gs_ffs, breaks = 10, labels = FALSE)

data$id <- 1:N

n.times <- data$interval

data_long <- data[rep(seq_len(N), times = n.times), ]

# create the correct intervals

for(i in unique(data_long$id)) {

n_length <- length(data_long$interval[data_long$id == i])

data_long$interval[data_long$id == i] <- 1:n_length

}

data_long$status <- vector(mode = "numeric",

length = nrow(data_long))

# put indication 1 on status at the interval that patient dies

for (i in 1:nrow(data_long)) {

if (data_long$gs_ffsstate[i] != data_long$status[i] &&

data_long$survival[i] == data_long$interval[i])

data_long$status[i] <- 1

}

intervals <- dummy_cols(as.factor(data_long$interval))

colnames(intervals) <- gsub(".data", "interval", colnames(intervals))

data_long <- data.frame(data_long, intervals[, 2:11])

return(data_long)

}

# function that creates data in the right long format for

# test set for each patient the interval goes from 1 year

# till 10 years

data_test_creator <- function(data){

N <- nrow(data)

# assign survival times to 10 intervals

data$interval <- max(as.numeric(cut(data$gs_ffs,

breaks = 10)))

# the true interval survival

data$survival <- as.numeric(cut(data$gs_ffs,

breaks = 10))

data$id <- 70001:(70000 + N) # define the patient ids abstractly

n.times <- data$interval

data_long <- data[rep(seq_len(N), times = n.times), ]

# create the correct intervals

for(i in unique(data_long$id)) {

n_length <- length(data_long$interval[data_long$id == i])

data_long$interval[data_long$id == i] <- 1:n_length

}

data_long$status <- vector(mode = "numeric",

length = nrow(data_long))

# put indication 1 on status at the intervals on

# which a patient has died

for (i in 1:nrow(data_long)) {

if (data_long$gs_ffsstate[i] == 1 &&

data_long$survival[i] <= data_long$interval[i])

data_long$status[i] <- 1

}

intervals2 <- dummy_cols(as.factor(data_long$interval))

colnames(intervals2) <- gsub(".data", "interval", colnames(intervals2))

data_long <- data.frame(data_long, intervals2[, 2:11])

return(data_long)

}

# function that calculates the metrics

measures_calculator <- function(trained_model,

datanew, real_status) {

df1 <- data.frame(hazard = predict_proba(trained_model,

as.matrix(datanew[, c(1:119, 126:135)]),

batch_size = 1000))

df1$id <- datanew$id # ids of the patients

df1$survival <- datanew$survival # survival time in years

groups <- split(df1, f = df1$id)

true_surv <- unlist(lapply(groups, function(x) {

surv_obj <- x$survival

true_res <- surv_obj[1]

return(true_res)}

))

group_probs <- lapply(groups, function(x) {

x <- cumprod(1 - x$hazard)})

pred_mat <- do.call("rbind", group_probs)

relative_probs <- vector(mode = "numeric",

length = length(group_probs))

for (i in 1:length(group_probs)){

temp <- group_probs[[i]]

ind <- which(1:10 == true_surv[i])

relative_probs[i] <- temp[ind]

}

N0 <- length(unique(df1$id)) # number of unique persons

# in the data frame create random id numbers

# to label the patients

df2 <- data.frame(relative_probs = relative_probs,

gs_ffs = true_surv,

gs_ffsstate = real_status,

id = (70001):(70000 + N0))

df2$prediction <- 1 - round(df2$relative_probs, digits = 0)

# create possible classes

classes <- c(0, 1)

# auxiliary confusion table

tabel <- table(factor(df2$prediction, levels = classes),

factor(df2$gs_ffsstate, levels = classes))

confusion_mat <- confusionMatrix(tabel, positive = "1")

accuracy <- sum(df2$prediction == df2$gs_ffsstate) / nrow(df2)

sensitivity <- sum(df2$gs_ffsstate == 1 & df2$prediction == 1) /

colSums(tabel)[2]

specificity <- sum(df2$gs_ffsstate == 0 & df2$prediction == 0) /

colSums(tabel)[1]

precision <- as.numeric(confusion_mat$byClass[5])

recall <- as.numeric(confusion_mat$byClass[6])

f1score <- as.numeric(confusion_mat$byClass[7])

brier_obj <- brier_nnet(prob_matrix = cbind(1, pred_mat), data = df2)

int_brier <- as.numeric(brier_obj$Int_brier)

brier_set <- brier_obj$Brier

all_weights <- get_weights(trained_model)

nr_weights <- length(unlist(all_weights))

# calculate variable importance

var_imp <- var_imp_keras(model = trained_model)

return(list(weights = nr_weights,

node_size = ncol(get_weights(trained_model)[[1]]),

cross_entropy =

result$metrics$loss[result$params$epochs],

accuracy = accuracy,

sensitivity = as.numeric(sensitivity),

specificity = as.numeric(specificity),

Precision = precision, Recall = recall,

F1score = f1score,

Integrated_brier = int_brier,

Brier_scores = brier_set,

Rel_imp = var_imp$Rels))

}

for (i in 1:nfolds) {

cat("Started iteration i = ", i, "\n")

indices <- folds[[i]]

cat("Creating the training set ...", "\n")

train_set <- data_train_creator(training_ffs_scaled[-indices, ]) # create the train set

cat("Creating the validation set ...", "\n")

validation_set <- data_test_creator(data = training_ffs_scaled[indices, ]) # create the validation set

# real status (if a patient survived or died) for the validation set

event_status <- training_ffs_scaled[indices, ]$gs_ffsstate

# create the matrices to be used for keras library

train_x <- as.matrix(train_set[, c(1:119, 126:135)]) # predictors: 119 variables + intervals

dimnames(train_x) <- NULL # the object must have empty dimnames

train_y <- train_set$status

validation_x <- as.matrix(validation_set[, c(1:119, 126:135)])

dimnames(validation_x) <- NULL # the object must have empty dimnames

validation_y <- validation_set$status

for (j in 1:nrow(combis)) {

cat("Testing combination number:", j, "of repeat", i, " out of 5", "\n")

cat("calculating for node size:", combis[j, 1], ", dropout rate:", combis[j, 2], "\n",

"and learning rate", combis[j, 3], "and weak class weight", combis[j, 4],

"and momentum", combis[j, 5], "...", "\n")

k_clear_session() # to avoid clutter from old models / layers in cross validation

# start building the model

fit_keras <- keras_model_sequential()

# Add layers to the model

# here we have logistic activation function for the inputs but also for the outputs

# we create a densely connected ANN to the output (input shape 119 + 10)

fit_keras %>%

layer_dense(units = combis[j, 1], activation = 'sigmoid', input_shape = c(129)) %>%

layer_dropout(rate = combis[j, 2]) %>%

layer_dense(units = 1, activation = 'sigmoid')

fit_keras %>% compile(

loss = 'binary_crossentropy', # for binary class classification problem

optimizer = optimizer_sgd(lr = combis[j, 3], momentum = combis[j, 5])

#, metrics = c("accuracy")

)

early_stopping <- callback_early_stopping(monitor = 'val_loss', patience = 5)

result <- fit_keras %>% fit(

train_x,

train_y,

epochs = 25,

batch_size = 1000,

validation_data = list(validation_x, validation_y),

class_weight = list("0" = 1, "1" = combis[j, 4]),

callbacks = c(early_stopping) # to enforce early stopping in case the loss function stops improving

)

# now that the model has run lets calulate the measures

# the total weights are 121*(node_size + bias_input) + (node_size + bias)*output

values <- measures_calculator(trained_model = fit_keras,

datanew = validation_set,

real_status = event_status)

#cv_error[i, j] <- values$cross_entropy # minimized cross-entropy

cv_weights[i, j] <- values$weights

cv_accuracy[i, j] <- round(values$accuracy, 3)

cv_sensitivity[i, j] <- round(values$sensitivity, 3)

cv_specificity[i, j] <- round(values$specificity, 3)

cv_precision[i, j] <- round(values$Precision, 3)

cv_recall[i, j] <- round(values$Recall, 3)

cv_f1score[i, j] <- round(values$F1score, 3)

cv_intbrier[i, j] <- round(values$Integrated_brier, 3)

cv_relimp[[i]][[j]] <- values$Rel_imp

# print(values$Brier_scores)

print(values$Integrated_brier)

}

}

df_sigmoid_ffs <- as.data.frame(cbind(node_size = combis[, 1],

dropout_rate = combis[, 2],

learning_rate = combis[, 3],

momentum = combis[, 5],

weak_weight = combis[, 4],

weights = colMeans(cv_weights),

#cross_entropy = colMeans(cv_error),

accuracy = colMeans(cv_accuracy),

sensitivity = colMeans(cv_sensitivity),

specificity = colMeans(cv_specificity),

precision = colMeans(cv_precision),

recall = colMeans(cv_recall),

f1score = colMeans(cv_f1score),

integrated_brier = colMeans(cv_intbrier)))

save(df_sigmoid_ffs, file = "results_sigmoid1h_ffs.RData")

save(cv_relimp, file = "results_cv_relimp1h_ffs.RData")

parallel::stopCluster(cl)

ind <- head(order(df_sigmoid_ffs$integrated_brier), 5)

df_sigmoid_ffs[ind, ]

# find the variable importance for the best combination on the training data

nr <- which.min(df_sigmoid_ffs$integrated_brier)

rel_imp_df <- rbind(cv_relimp[[1]][[nr]], cv_relimp[[2]][[nr]],

cv_relimp[[3]][[nr]], cv_relimp[[4]][[nr]],

cv_relimp[[5]][[nr]])

rel_imp <- colMeans(rel_imp_df)

df <- data.frame(name = colnames(training_ffs_long)[c(1:119, 126:135)],

var_imp = rel_imp)

df <- df[order(df$var_imp, decreasing = TRUE), ]

rownames(df) <- NULL

# separately for variables without time intervals

df2 <- df[!df$name %in% colnames(training_ffs_long)[126:135], ]

df2 <- df2[order(df2$var_imp, decreasing = TRUE), ]

rownames(df2) <- NULL

head(df2, 10)

df3 <- df[df$name %in% colnames(training_ffs_long)[126:135], ]

df3 <- df3[order(df3$var_imp, decreasing = TRUE), ]

rownames(df3) <- NULL

df3

####################################################################

# Neural networks: training example for 2 hidden layer

####################################################################

install_packages <- c("fastDummies", "keras", "survival", "pec",

"caret", "doParallel")

for (i in 1:length (install_packages)){

if (!install_packages[i] %in% installed.packages()){

install.packages(install_packages[i])

}

}

# Survival neural networks

library(fastDummies)

library(keras)

library(survival)

library(pec)

library(caret)

library(doParallel)

cl <- makeCluster(2)

registerDoParallel(cl)

# read the scaled training data for ffs (this is the scaled short format)

load("training_ffs_scaled.RData")

load("test_ffs_scaled.RData")

load("training_ffs_long.RData")

load("test_ffs_long.RData")

# load file the functions

source("the_functions2.R")

#######################################################################################################

#######################################################################################################

# set up the cross-validation

# most popular optimization algorithms used are the Stochastic Gradient Descent (SGD), ADAM and RMSprop

# you need to tune certain parameters such as learning rate or momentum

nfolds <- 5

set.seed(1234)

folds <- createFolds(training_ffs_scaled$gs_ffs, k = 5, list = TRUE)

# node_size <- seq(10, 130, by = 20) # grid of node sizes

# dropout_rate <- c(0.1, 0.2, 0.3)

# lr <- c(0.01, 0.1, 0.2)

# class_weights <- c(1, 2.236) # increase the weight of weak class according to 69.1 / 30.9

# momentum <- c(0.8, 0.9)

# combis <- expand.grid(node_size, dropout_rate, lr, class_weights, momentum)

node_size <- 110 # grid of node sizes

dropout_rate <- 0.3

lr <- 0.2

class_weights <- 1 # increase the weight of weak class according to 69.1 / 30.9

momentum <- 0.9

combis <- expand.grid(node_size, dropout_rate, lr, class_weights, momentum)

# initialize objects

# cv_error <- matrix(0, nrow = nfolds, ncol = nrow(combis))

cv_accuracy <- matrix(0, nrow = nfolds, ncol = nrow(combis))

cv_specificity <- matrix(0, nrow = nfolds, ncol = nrow(combis))

cv_sensitivity <- matrix(0, nrow = nfolds, ncol = nrow(combis))

cv_precision <- matrix(0, nrow = nfolds, ncol = nrow(combis))

cv_recall <- matrix(0, nrow = nfolds, ncol = nrow(combis))

cv_f1score <- matrix(0, nrow = nfolds, ncol = nrow(combis))

cv_weights <- matrix(0, nrow = nfolds, ncol = nrow(combis))

cv_intbrier <- matrix(0, nrow = nfolds, ncol = nrow(combis))

cv_relimp <- list(vector(mode = "list", length = nrow(combis)),

vector(mode = "list", length = nrow(combis)),

vector(mode = "list", length = nrow(combis)),

vector(mode = "list", length = nrow(combis)),

vector(mode = "list", length = nrow(combis)))

data_train_creator <- function(data){

N <- nrow(data)

# assign survival times to 10 intervals, each is a 1-year period

data$interval <- cut(data$gs_ffs, breaks = 10, labels = FALSE)

data$survival <- cut(data$gs_ffs, breaks = 10, labels = FALSE)

data$id <- 1:N

n.times <- data$interval

data_long <- data[rep(seq_len(N), times = n.times), ]

# create the correct intervals

for(i in unique(data_long$id)) {

n_length <- length(data_long$interval[data_long$id == i])

data_long$interval[data_long$id == i] <- 1:n_length

}

data_long$status <- vector(mode = "numeric",

length = nrow(data_long))

# put indication 1 on status at the interval that patient dies

for (i in 1:nrow(data_long)) {

if (data_long$gs_ffsstate[i] != data_long$status[i] &&

data_long$survival[i] == data_long$interval[i])

data_long$status[i] <- 1

}

intervals <- dummy_cols(as.factor(data_long$interval))

colnames(intervals) <- gsub(".data", "interval", colnames(intervals))

data_long <- data.frame(data_long, intervals[, 2:11])

return(data_long)

}

# function that creates data in the right long format for

# test set for each patient the interval goes from 1 year

# till 10 years

data_test_creator <- function(data){

N <- nrow(data)

# assign survival times to 10 intervals

data$interval <- max(as.numeric(cut(data$gs_ffs,

breaks = 10)))

# the true interval survival

data$survival <- as.numeric(cut(data$gs_ffs,

breaks = 10))

data$id <- 70001:(70000 + N) # define the patient ids abstractly

n.times <- data$interval

data_long <- data[rep(seq_len(N), times = n.times), ]

# create the correct intervals

for(i in unique(data_long$id)) {

n_length <- length(data_long$interval[data_long$id == i])

data_long$interval[data_long$id == i] <- 1:n_length

}

data_long$status <- vector(mode = "numeric",

length = nrow(data_long))

# put indication 1 on status at the intervals on

# which a patient has died

for (i in 1:nrow(data_long)) {

if (data_long$gs_ffsstate[i] == 1 &&

data_long$survival[i] <= data_long$interval[i])

data_long$status[i] <- 1

}

intervals2 <- dummy_cols(as.factor(data_long$interval))

colnames(intervals2) <- gsub(".data", "interval", colnames(intervals2))

data_long <- data.frame(data_long, intervals2[, 2:11])

return(data_long)

}

# function that calculates the metrics

measures_calculator <- function(trained_model,

datanew, real_status) {

df1 <- data.frame(hazard = predict_proba(trained_model,

as.matrix(datanew[, c(1:119, 126:135)]),

batch_size = 1000))

df1$id <- datanew$id # ids of the patients

df1$survival <- datanew$survival # survival time in years

groups <- split(df1, f = df1$id)

true_surv <- unlist(lapply(groups, function(x) {

surv_obj <- x$survival

true_res <- surv_obj[1]

return(true_res)}

))

group_probs <- lapply(groups, function(x) {

x <- cumprod(1 - x$hazard)})

pred_mat <- do.call("rbind", group_probs)

relative_probs <- vector(mode = "numeric",

length = length(group_probs))

for (i in 1:length(group_probs)){

temp <- group_probs[[i]]

ind <- which(1:10 == true_surv[i])

relative_probs[i] <- temp[ind]

}

N0 <- length(unique(df1$id)) # number of unique persons

# in the data frame create random id numbers

# to label the patients

df2 <- data.frame(relative_probs = relative_probs,

gs_ffs = true_surv,

gs_ffsstate = real_status,

id = (70001):(70000 + N0))

df2$prediction <- 1 - round(df2$relative_probs, digits = 0)

# create possible classes

classes <- c(0, 1)

# auxiliary confusion table

tabel <- table(factor(df2$prediction, levels = classes),

factor(df2$gs_ffsstate, levels = classes))

confusion_mat <- confusionMatrix(tabel, positive = "1")

accuracy <- sum(df2$prediction == df2$gs_ffsstate) / nrow(df2)

sensitivity <- sum(df2$gs_ffsstate == 1 & df2$prediction == 1) /

colSums(tabel)[2]

specificity <- sum(df2$gs_ffsstate == 0 & df2$prediction == 0) /

colSums(tabel)[1]

precision <- as.numeric(confusion_mat$byClass[5])

recall <- as.numeric(confusion_mat$byClass[6])

f1score <- as.numeric(confusion_mat$byClass[7])

brier_obj <- brier_nnet(prob_matrix = cbind(1, pred_mat), data = df2)

int_brier <- as.numeric(brier_obj$Int_brier)

brier_set <- brier_obj$Brier

all_weights <- get_weights(trained_model)

nr_weights <- length(unlist(all_weights))

# calculate variable importance

var_imp <- var_imp_keras2h(model = trained_model)

return(list(weights = nr_weights,

node_size = ncol(get_weights(trained_model)[[1]]),

cross_entropy =

result$metrics$loss[result$params$epochs],

accuracy = accuracy,

sensitivity = as.numeric(sensitivity),

specificity = as.numeric(specificity),

Precision = precision, Recall = recall,

F1score = f1score,

Integrated_brier = int_brier,

Brier_scores = brier_set,

Rel_imp = var_imp$Rels))

}

for (i in 1:nfolds) {

cat("Started iteration i = ", i, "\n")

indices <- folds[[i]]

cat("Creating the training set ...", "\n")

train_set <- data_train_creator(training_ffs_scaled[-indices, ]) # create the train set

cat("Creating the validation set ...", "\n")

validation_set <- data_test_creator(data = training_ffs_scaled[indices, ]) # create the validation set

# real status (if a patient survived or died) for the validation set

event_status <- training_ffs_scaled[indices, ]$gs_ffsstate

# create the matrices to be used for keras library

train_x <- as.matrix(train_set[, c(1:119, 126:135)]) # predictors: 119 variables + intervals

dimnames(train_x) <- NULL # the object must have empty dimnames

train_y <- train_set$status

validation_x <- as.matrix(validation_set[, c(1:119, 126:135)])

dimnames(validation_x) <- NULL # the object must have empty dimnames

validation_y <- validation_set$status

for (j in 1:nrow(combis)) {

cat("Testing combination number:", j, "of repeat", i, " out of 5", "\n")

cat("calculating for node size:", combis[j, 1], ", dropout rate:", combis[j, 2], "\n",

"and learning rate", combis[j, 3], "and weak class weight", combis[j, 4],

"and momentum", combis[j, 5], "...", "\n")

k_clear_session() # to avoid clutter from old models / layers in cross validation

# start building the model

fit_keras <- keras_model_sequential()

# Add layers to the model

# here we have logistic activation function for the inputs but also for the outputs

# we create a densely connected ANN to the output (input shape 119 + 10)

fit_keras %>%

layer_dense(units = combis[j, 1], activation = 'sigmoid', input_shape = c(129)) %>%

layer_dropout(rate = combis[j, 2]) %>%

layer_dense(units = combis[j, 1], activation = 'sigmoid') %>%

layer_dropout(rate = combis[j, 2]) %>%

layer_dense(units = 1, activation = 'sigmoid')

fit_keras %>% compile(

loss = 'binary_crossentropy', # for binary class classification problem

optimizer = optimizer_sgd(lr = combis[j, 3], momentum = combis[j, 5])

#, metrics = c("accuracy")

)

early_stopping <- callback_early_stopping(monitor = 'val_loss', patience = 5)

result <- fit_keras %>% fit(

train_x,

train_y,

epochs = 25,

batch_size = 1000,

validation_data = list(validation_x, validation_y),

class_weight = list("0" = 1, "1" = combis[j, 4]),

callbacks = c(early_stopping) # to enforce early stopping in case the loss function stops improving

)

# now that the model has run lets calulate the measures

# the total weights are 121*(node_size + bias_input) + (node_size + bias)*output

values <- measures_calculator(trained_model = fit_keras,

datanew = validation_set,

real_status = event_status)

#cv_error[i, j] <- values$cross_entropy # minimized cross-entropy

cv_weights[i, j] <- values$weights

cv_accuracy[i, j] <- round(values$accuracy, 3)

cv_sensitivity[i, j] <- round(values$sensitivity, 3)

cv_specificity[i, j] <- round(values$specificity, 3)

cv_precision[i, j] <- round(values$Precision, 3)

cv_recall[i, j] <- round(values$Recall, 3)

cv_f1score[i, j] <- round(values$F1score, 3)

cv_intbrier[i, j] <- round(values$Integrated_brier, 3)

cv_relimp[[i]][[j]] <- values$Rel_imp

# print(values$Brier_scores)

print(values$Integrated_brier)

}

}

df_sigmoid_ffs <- as.data.frame(cbind(node_size = combis[, 1],

dropout_rate = combis[, 2],

learning_rate = combis[, 3],

momentum = combis[, 5],

weak_weight = combis[, 4],

weights = colMeans(cv_weights),

#cross_entropy = colMeans(cv_error),

accuracy = colMeans(cv_accuracy),

sensitivity = colMeans(cv_sensitivity),

specificity = colMeans(cv_specificity),

precision = colMeans(cv_precision),

recall = colMeans(cv_recall),

f1score = colMeans(cv_f1score),

integrated_brier = colMeans(cv_intbrier)))

save(df_sigmoid_ffs, file = "results_sigmoid2h_ffs.RData")

save(cv_relimp, file = "results_cv_relimp2h_ffs.RData")

parallel::stopCluster(cl)

ind <- head(order(df_sigmoid_ffs$integrated_brier), 5)

df_sigmoid_ffs[ind, ]

# find the variable importance for the best combination on the training data

nr <- which.min(df_sigmoid_ffs$integrated_brier)

rel_imp_df <- rbind(cv_relimp[[1]][[nr]], cv_relimp[[2]][[nr]],

cv_relimp[[3]][[nr]], cv_relimp[[4]][[nr]],

cv_relimp[[5]][[nr]])

rel_imp <- colMeans(rel_imp_df)

df <- data.frame(name = colnames(training_ffs_long)[c(1:119, 126:135)],

var_imp = rel_imp)

df <- df[order(df$var_imp, decreasing = TRUE), ]

rownames(df) <- NULL

# separately for variables without time intervals

df2 <- df[!df$name %in% colnames(training_ffs_long)[126:135], ]

df2 <- df2[order(df2$var_imp, decreasing = TRUE), ]

rownames(df2) <- NULL

head(df2, 10)

df3 <- df[df$name %in% colnames(training_ffs_long)[126:135], ]

df3 <- df3[order(df3$var_imp, decreasing = TRUE), ]

rownames(df3) <- NULL

df3

####################################################################

# Neural networks: predicting on the test data

####################################################################

install_packages <- c("fastDummies", "keras", "survival", "pec",

"caret", "ggpubr")

for (i in 1:length (install_packages)){

if (!install_packages[i] %in% installed.packages()){

install.packages(install_packages[i])

}

}

# predictions on test data for survival neural networks: new

library(fastDummies)

library(keras)

library(survival)

library(pec)

library(caret)

library(ggpubr)

# library(doParallel)

# cl <- makeCluster(4)

# registerDoParallel(cl)

use_session_with_seed(seed = 12345, disable_gpu = TRUE,

disable_parallel_cpu = TRUE,

quiet = FALSE)

source("the_functions2.R")

load("training_ffs_scaled.Rdata")

load("test_ffs_scaled.Rdata")

# load the already created long format of the training and the test data

load("training_ffs_long.Rdata")

load("test_ffs_long.Rdata")

measures_calculator <- function(trained_model,

datanew, real_status) {

df1 <- data.frame(hazard = predict_proba(trained_model,

as.matrix(datanew[, c(1:119, 126:135)]),

batch_size = 1000))

df1$id <- datanew$id # ids of the patients

df1$survival <- datanew$survival # survival time in years

groups <- split(df1, f = df1$id)

true_surv <- unlist(lapply(groups, function(x) {

surv_obj <- x$survival

true_res <- surv_obj[1]

return(true_res)}

))

group_probs <- lapply(groups, function(x) {

x <- cumprod(1 - x$hazard)})

pred_mat <- do.call("rbind", group_probs)

relative_probs <- vector(mode = "numeric",

length = length(group_probs))

for (i in 1:length(group_probs)){

temp <- group_probs[[i]]

ind <- which(1:10 == true_surv[i])

relative_probs[i] <- temp[ind]

}

N0 <- length(unique(df1$id)) # number of unique persons

# in the data frame create random id numbers

# to label the patients

df2 <- data.frame(relative_probs = relative_probs,

gs_ffs = true_surv,

gs_ffsstate = real_status,

id = (70001):(70000 + N0))

df2$prediction <- 1 - round(df2$relative_probs, digits = 0)

# create possible classes

classes <- c(0, 1)

# auxiliary confusion table

tabel <- table(factor(df2$prediction, levels = classes),

factor(df2$gs_ffsstate, levels = classes))

confusion_mat <- confusionMatrix(tabel, positive = "1")

accuracy <- sum(df2$prediction == df2$gs_ffsstate) / nrow(df2)

sensitivity <- sum(df2$gs_ffsstate == 1 & df2$prediction == 1) /

colSums(tabel)[2]

specificity <- sum(df2$gs_ffsstate == 0 & df2$prediction == 0) /

colSums(tabel)[1]

precision <- as.numeric(confusion_mat$byClass[5])

recall <- as.numeric(confusion_mat$byClass[6])

f1score <- as.numeric(confusion_mat$byClass[7])

brier_obj <- brier_nnet(prob_matrix = cbind(1, pred_mat), data = df2)

int_brier <- as.numeric(brier_obj$Int_brier)

brier_set <- brier_obj$Brier

all_weights <- get_weights(trained_model)

nr_weights <- length(unlist(all_weights))

return(list(weights = nr_weights,

node_size = ncol(get_weights(trained_model)[[1]]),

cross_entropy =

result$metrics$loss[result$params$epochs],

accuracy = accuracy,

sensitivity = as.numeric(sensitivity),

specificity = as.numeric(specificity),

Precision = precision, Recall = recall,

F1score = f1score,

Integrated_brier = int_brier,

Brier_scores = brier_set))

}

# model with one hidden layer

fit_keras <- keras_model_sequential()

# Add layers to the model

# here we have logistic activation function for the inputs but also for the outputs

# we create a densely connected ANN to the output

fit_keras %>%

layer_dense(units = 85, activation = 'sigmoid', input_shape = c(129)) %>%

layer_dropout(rate = 0.2) %>%

layer_dense(units = 1, activation = 'sigmoid')

fit_keras %>% compile(

loss = 'binary_crossentropy', # for binary class classification problem

optimizer = optimizer_sgd(lr = 0.2, momentum = 0.9)

#, metrics = c("accuracy")

)

early_stopping <- callback_early_stopping(monitor = 'val_loss', patience = 5)

event_status <- test_ffs_scaled$gs_ffsstate

# create the matrices to be used for keras library

train_x <- as.matrix(training_ffs_long[, c(1:119, 126:135)])

dimnames(train_x) <- NULL # the object must have empty dimnames

train_y <- training_ffs_long$status

test_x <- as.matrix(validation_ffs_long[, c(1:119, 126:135)])

dimnames(test_x) <- NULL # the object must have empty dimnames

test_y <- validation_ffs_long$status

result <- fit_keras %>% fit(

train_x,

train_y,

epochs = 25,

batch_size = 1000,

validation_data = list(test_x, test_y),

class_weight = list("0" = 1, "1" = 1),

callbacks = c(early_stopping)

)

metrics_model_nn1h <- measures_calculator(trained_model = fit_keras,

datanew = test_ffs_long,

real_status = event_status)

metrics_model_nn1h$weights

plot(0:10, metrics_model_nn1h$Brier_scores, type = "l", ylim = c(0, 0.33),

xlab = "Time since transplantation in years",

ylab = "Prediction error (Brier score)")

brier_nn1h <- metrics_model_nn1h$Brier_scores

save(brier_nn1h, file = "brier_nn1h.RData")

#####################################################################################

# Calculating the VIMP for a neural network with 1 hidden layer on the test data

var_imp <- var_imp_keras(model = fit_keras)

df <- data.frame(name = var_imp$Names, var_imp = var_imp$Rels)

df <- df[order(df$var_imp, decreasing = TRUE), ]

rownames(df) <- NULL

# separately for variables without time intervals

df2 <- df[!df$name %in% colnames(training_ffs_long)[126:135], ]

df2 <- df2[order(df2$var_imp, decreasing = TRUE), ]

rownames(df2) <- NULL

head(df2, 10)

plot1 <- ggbarplot(data = df2[1:5, ],

x = "name", y = "var_imp",

fill = rainbow(5)) +

xlab("Variable name") +

ylab("Relative importance") +

ylim(c(0, 0.04)) +

theme(axis.text=element_text(size = 7),

axis.title=element_text(size=14, face="bold"))

plot1 # plotting the 5 most important variables

df3 <- df[df$name %in% colnames(training_ffs_long)[126:135], ]

df3 <- df3[order(df3$var_imp, decreasing = TRUE), ]

rownames(df3) <- NULL

df3

##############################################################################

use_session_with_seed(seed = 12345, disable_gpu = TRUE,

disable_parallel_cpu = TRUE,

quiet = FALSE)

# model with 2 hidden layers

# find the metrics for combination node_size = 100, dropout rate = 0.2,

# learning rate 0.1, momentum 0.9, weak class weight 1

fit_keras2 <- keras_model_sequential()

# Add layers to the model

# here we have logistic activation function for the inputs but also for the outputs

# we create a densely connected ANN to the output

fit_keras2 %>%

layer_dense(units = 110, activation = 'sigmoid', input_shape = c(129)) %>%

layer_dropout(rate = 0.1) %>%

layer_dense(units = 110, activation = 'sigmoid') %>%

layer_dropout(rate = 0.1) %>%

layer_dense(units = 1, activation = 'sigmoid')

fit_keras2 %>% compile(

loss = 'binary_crossentropy', # for binary class classification problem

optimizer = optimizer_sgd(lr = 0.2, momentum = 0.9)

)

early_stopping <- callback_early_stopping(monitor = 'val_loss', patience = 5)

event_status <- test_ffs_scaled$gs_ffsstate

# create the matrices to be used for keras library

train_x <- as.matrix(training_ffs_long[, c(1:119, 126:135)]) # predictors: 119 variables + interval

dimnames(train_x) <- NULL # the object must have empty dimnames

train_y <- training_ffs_long$status

test_x <- as.matrix(validation_ffs_long[, c(1:119, 126:135)])

dimnames(test_x) <- NULL # the object must have empty dimnames

test_y <- validation_ffs_long$status

result <- fit_keras2 %>% fit(

train_x,

train_y,

epochs = 25,

batch_size = 1000,

validation_data = list(test_x, test_y),

class_weight = list("0" = 1, "1" = 1),

callbacks = c(early_stopping)

)

metrics_model_nn2h <- measures_calculator(trained_model = fit_keras2,

datanew = test_ffs_long,

real_status = event_status)

metrics_model_nn2h$weights

brier_nn2h <- metrics_model_nn2h$Brier_scores

save(brier_nn2h, file = "brier_nn2h.RData")

plot(0:10, metrics_model_nn2h$Brier_scores, type = "l", ylim = c(0, 0.33),

xlab = "Time since transplantation in years",

ylab = "Prediction error (Brier score)")

#####################################################################################

# Calculating the VIMP for a neural network with 2 hidden layers on the test data

var_imp2 <- var_imp_keras2h(model = fit_keras2)

dfb <- data.frame(name = var_imp2$Names, var_imp = var_imp2$Rels)

dfb <- dfb[order(dfb$var_imp, decreasing = TRUE), ]

rownames(dfb) <- NULL

# separately for variables without time intervals

dfb2 <- dfb[!dfb$name %in% colnames(training_ffs_long)[126:135], ]

dfb2 <- dfb2[order(dfb2$var_imp, decreasing = TRUE), ]

rownames(dfb2) <- NULL

head(dfb2, 10)

plot2 <- ggbarplot(data = dfb2[1:5, ],

x = "name", y = "var_imp",

fill = rainbow(5)) +

xlab("Variable name") +

ylab("Relative importance") +

ylim(c(0, 0.04)) +

theme(axis.text=element_text(size = 7),

axis.title=element_text(size=14, face="bold"))

plot2 # plotting the 5 most important variables

dfb3 <- dfb[dfb$name %in% colnames(training_ffs_long)[126:135], ]

dfb3 <- dfb3[order(dfb3$var_imp, decreasing = TRUE), ]

rownames(dfb3) <- NULL

dfb3

####################################################################

# Comparisons between the models: create synthetic patients

####################################################################

# create synthetic datasets

load("training_ffs.RData")

load("training_ffs_scaled.RData")

# make a function that identifies the nature of your variable

most_common <- function(x) {

if (is.numeric(x)) return(median(x))

else {

tx <- table(x)

m <- which(tx == max(tx))[1]

fac <- factor(names(m), levels = levels(x))

return(fac)

}

}

vars <- colnames(training_ffs)[1:97]

freqa <- lapply(vars, function(x) most_common(training_ffs[, x]))

df_synth_normal <- do.call(cbind.data.frame, freqa)

colnames(df_synth_normal) <- vars

df_synth_normal <- df_synth_normal[rep(seq_len(1), times = 6), ]

str(df_synth_normal)

save(df_synth_normal, file = "df_synth_normal.RData")

vars2 <- colnames(training_ffs_scaled)[1:119]

freqb <- lapply(vars2, function(x) most_common(training_ffs_scaled[, x]))

df_synth_scaled <- do.call(cbind.data.frame, freqb)

colnames(df_synth_scaled) <- vars2

df_synth_scaled <- df_synth_scaled[rep(seq_len(1), times = 6), ]

str(df_synth_scaled)

save(df_synth_scaled, file = "df_synth_scaled.RData")

####################################################################

# Comparisons between the models: on predictive performance + on synthetic patients

####################################################################

install_packages <- c("glmnet", "pec", "survival", "caret",

"ggplot2", "gridExtra", "hdnom", "keras",

"fastDummies", "randomForestSRC")

for (i in 1:length (install_packages)){

if (!install_packages[i] %in% installed.packages()){

install.packages(install_packages[i])

}

}

# comparisons between the methods

library(glmnet)

library(pec)

library(survival)

library(caret)

library(survcomp)

library(ggplot2)

library(gridExtra)

library(hdnom)

library(keras)

library(fastDummies)

library(randomForestSRC)

library(parallel)

options(rf.cores = 60, mc.cores = 60)

source("the_functions2.R")

load("training_ffs.RData")

#load("training_ffs_extended.RData")

load("training_ffs_scaled.RData")

load("training_ffs_long.RData")

load("test_ffs.RData")

#load("test_ffs_extended.RData")

load("test_ffs_scaled.RData")

load("test_ffs_long.RData")

surv_f <- Surv(gs_ffs, gs_ffsstate) ~ 1

# Cox full

cox_full <- coxph(Surv(gs_ffs, gs_ffsstate) ~.,

data = training_ffs, x = TRUE, y = TRUE)

summ <- summary(cox_full)

# coefficients from the Cox model

res_nolist <- unlist(summ$coefficients)

# coefficients from the Cox model sorted by Z-score value

round(res_nolist[order(res_nolist[, 4], decreasing = T), ][1:12, ], digits = 3)

pi_cox_full <- predict(cox_full, newdata = test_ffs)

cindex_cox_full <- round(concordance.index(x = pi_cox_full,

surv.time = test_ffs$gs_ffs,

surv.event = test_ffs$gs_ffsstate)$c.index,

digits = 4)

# Cox backward

# model cox backward

cox_back <- selectCox(formula = Surv(gs_ffs, gs_ffsstate) ~.,

data = training_ffs, rule = "aic")

vars_back <- cox_back$In # variables

f_back <- as.formula(paste("Surv(gs_ffs, gs_ffsstate) ~",

paste(vars_back, collapse = "+")))

# Cox backward in coxph object

cox_back <- coxph(formula = f_back, x = TRUE, y = TRUE,

data = training_ffs, method = "breslow")

pi_cox_back <- predict(cox_back, newdata = test_ffs)

cindex_cox_back <- round(concordance.index(x = pi_cox_back,

surv.time = test_ffs$gs_ffs,

surv.event = test_ffs$gs_ffsstate)$c.index,

digits = 4)

# attr(cox_back$coefficients, "names")

# Cox LASSO

response_pair <- c("gs_ffs", "gs_ffsstate")

# for the training set

X_train <- training_ffs[, !(colnames(training_ffs) %in% response_pair)]

X_train_ffs <- model.matrix(~., X_train)[, -1] # create martix X for glmnet

# dim(X_train_ffs) # 41530 x 119

Y_train_ffs <- training_ffs[, colnames(training_ffs) %in% response_pair] # create matrix Y

Y_survtrain_ffs <- Surv(Y_train_ffs$gs_ffs, Y_train_ffs$gs_ffsstate)

# for the test set

x_test <- test_ffs[, !(colnames(test_ffs) %in% response_pair)]

X_test_ffs <- model.matrix(~., x_test)[, -1]

Y_test_ffs <- test_ffs[, colnames(test_ffs) %in% response_pair]

# Surv function packages survival data into the form expected by glmnet

Y_survtest_ffs <- Surv(Y_test_ffs$gs_ffs, Y_test_ffs$gs_ffsstate)

# use these two datasets to fit Cox model in the selected features

training_ffs_extended <- as.data.frame(cbind(X_train_ffs, Y_train_ffs))

test_ffs_extended <- as.data.frame(cbind(X_test_ffs, Y_test_ffs))

set.seed(12345)

folds <- createFolds(y = training_ffs$gs_ffs, k = 5, list = FALSE)

cv.fit <- cv.glmnet(x = X_train_ffs, y = Y_survtrain_ffs,

foldid = folds, parallel = TRUE,

family = "cox", grouped = TRUE,

maxit = 1000)

# Fit a generalized linear model via penalized maximum likelihood,

# alpha = 1 means apply LASSO penalty

fit <- glmnet(x = X_train_ffs, y = Y_survtrain_ffs, alpha = 1,

lambda = cv.fit$lambda.1se,

family = "cox", maxit = 1000)

# returns the p length coef vector of the solution

# coefficients <- coef(fit, s = cv.fit$lambda.1se)

# # available coefficients for test set, the same will be non-zero as the train set

# names_coefs <- unlist(coefficients@Dimnames[1])

# # corresponding to lambda = cv.fit$lambda.min.

# active_index <- which(coefficients != 0)

# active_coefs <- coefficients[active_index]

# vars_active_1se <- names_coefs[active_index]

pi_cox_lasso <- predict(cv.fit, newx = X_test_ffs,

s = "lambda.1se", type="link") # gives linear predictors - same as lp

cindex_cox_lasso <- round(concordance.index(x = pi_cox_lasso,

surv.time = test_ffs_extended$gs_ffs,

surv.event = test_ffs_extended$gs_ffsstate)$c.index,

digits = 4)

# calculate the Brier scores

cox_pair <- as.data.frame(cbind(test_ffs_extended$gs_ffs,

test_ffs_extended$gs_ffsstate))

colnames(cox_pair) <- c("time", "status")

times <- seq(0, 10, length.out = 11)

# find the probabilities of the models

cox_probs_full <- predictSurvProb(cox_full, test_ffs,

times = times)

brier_cox_full <- brier_general_new(prob_matrix = cox_probs_full,

data = test_ffs,

times = times)

cox_probs_back <- predictSurvProb(cox_back, test_ffs,

times = times)

brier_cox_back <- brier_general_new(prob_matrix = cox_probs_back,

data = test_ffs,

times = times)

response_pair <- data.frame(time = test_ffs$gs_ffs, status = test_ffs$gs_ffsstate)

# function to estimate survival probabilities from a glmnet object

predictProb_glmnet <- function (glmnet_object, response_pair, x, times)

{

lp <- as.numeric(predict(glmnet_object, newx = data.matrix(x),

s = "lambda.1se", type="link"))

basesurv <- glmnet_basesurv(time = response_pair$time,

event = response_pair$status,

lp = lp, times.eval = times)

p <- exp(exp(lp) %*% -t(basesurv$cumulative_base_hazard))

if (NROW(p) != NROW(x) || NCOL(p) != length(times))

stop("Prediction failed")

return(p)

}

probs_lasso <- predictProb_glmnet(glmnet_object = cv.fit,

response_pair = response_pair,

x = X_test_ffs,

times = seq(0, 10, length.out = 11))

brier_cox_lasso <- brier_general_new(prob_matrix = probs_lasso,

data = test_ffs_extended,

times = times)

# for the random survival forest

load("probs_rsf.RData")

brier_rsf <- brier_general_new(prob_matrix = probs_rsf,

data = test_ffs,

times = seq(0, 10, length.out = 11))

# model_rsf <- rfsrc(Surv(gs_ffs, gs_ffsstate) ~ ., splitrule = "logrank", nsplit = 5,

# data = training_ffs, ntree = 300, split.depth = "all.trees",

# var.used = "all.trees", seed = -12345, mtry = 12, nodesize = 50,

# sampsize = nrow(training_ffs) / 2, forest = TRUE, importance = TRUE

# )

# fit_test <- predict(model_rsf, newdata = test_ffs, seed = -12345,

# split.depth = "all.trees", forest = FALSE)

# cindex_rsf <- 1 - fit_test$err.rate[fit_test$ntree]

cindex_rsf <- 0.622

load("brier_rsf.RData")

load("brier_nn1h.RData")

load("brier_nn2h.RData")

# create prediction error plot

line1 <- data.frame(times = brier_cox_full$Time,

pe = brier_cox_full$Brier,

Model = "Cox all variables")

line2 <- data.frame(times = brier_cox_back$Time,

pe = brier_cox_back$Brier,

Model = "Cox backward")

line3 <- data.frame(times = brier_cox_lasso$Time,

pe = brier_cox_lasso$Brier,

Model = "Cox LASSO")

line4 <- data.frame(times = times,

pe = brier_rsf,

Model = "RSF")

line5 <- data.frame(times = times,

pe = brier_nn1h,

Model = "NN 1 hidden")

line6 <- data.frame(times = times,

pe = brier_nn2h,

Model = "NN 2 hidden")

df <- rbind(line1, line2, line3, line4, line5, line6)

ggplot(df, aes(x = times, y = pe, color = Model)) +

geom_line(size = 0.9) +

xlab("Time in years since transplantation") +

ylab("Prediction error (Brier score)") +

scale_x_continuous(breaks = 0:10, limits = c(0, 10)) +

ylim(c(0, 0.25)) +

theme_classic() +

ggtitle(" ") +

theme(plot.title = element_text(hjust = 0.5)) +

scale_color_manual(values=c("black", "red", "purple",

"blue", "green", "darkgreen"))

# scale_colour_grey(start = 1, end = 0.3, na.value = "red",

# aesthetics = "colour")

# survival curves for new hypothetical patients

# candidate variables are retransplantation, donorage and lifesupportort

##############################################################################

cox_full <- coxph(Surv(gs_ffs, gs_ffsstate) ~.,

data = training_ffs, x = TRUE, y = TRUE)

use_session_with_seed(seed = 12345, disable_gpu = TRUE,

disable_parallel_cpu = TRUE,

quiet = FALSE)

# run a neural network with 1 hidden layer

# function that creates data in the right long format for

# test set for each patient the interval goes from 1 year

# till 10 years

# function that creates data in the right long format for

# test set for each patient the interval goes from 1 year

# till 10 years

data_test_creator <- function(data){

N <- nrow(data)

# assign survival times to 10 intervals

data$interval <- max(as.numeric(cut(data$gs_ffs,

breaks = 10)))

# the true interval survival

data$survival <- as.numeric(cut(data$gs_ffs,

breaks = 10))

data$id <- 70001:(70000 + N) # define the patient ids abstractly

n.times <- data$interval

data_long <- data[rep(seq_len(N), times = n.times), ]

# create the correct intervals

for(i in unique(data_long$id)) {

n_length <- length(data_long$interval[data_long$id == i])

data_long$interval[data_long$id == i] <- 1:n_length

}

data_long$status <- vector(mode = "numeric",

length = nrow(data_long))

# put indication 1 on status at the intervals on

# which a patient has died

for (i in 1:nrow(data_long)) {

if (data_long$gs_ffsstate[i] == 1 &&

data_long$survival[i] <= data_long$interval[i])

data_long$status[i] <- 1

}

intervals2 <- dummy_cols(as.factor(data_long$interval))

colnames(intervals2) <- gsub(".data", "interval", colnames(intervals2))

data_long <- data.frame(data_long, intervals2[, 2:11])

return(data_long)

}

measures_calculator <- function(trained_model,

datanew, real_status) {

df1 <- data.frame(hazard = predict_proba(trained_model,

as.matrix(datanew[, c(1:119, 126:135)]),

batch_size = 1000))

df1$id <- datanew$id # ids of the patients

df1$survival <- datanew$survival # survival time in years

groups <- split(df1, f = df1$id)

true_surv <- unlist(lapply(groups, function(x) {

surv_obj <- x$survival

true_res <- surv_obj[1]

return(true_res)}

))

group_probs <- lapply(groups, function(x) {

x <- cumprod(1 - x$hazard)})

pred_mat <- do.call("rbind", group_probs)

relative_probs <- vector(mode = "numeric",

length = length(group_probs))

for (i in 1:length(group_probs)){

temp <- group_probs[[i]]

ind <- which(1:10 == true_surv[i])

relative_probs[i] <- temp[ind]

}

N0 <- length(unique(df1$id)) # number of unique persons

# in the data frame create random id numbers

# to label the patients

df2 <- data.frame(relative_probs = relative_probs,

gs_ffs = true_surv,

gs_ffsstate = real_status,

id = (70001):(70000 + N0))

df2$prediction <- 1 - round(df2$relative_probs, digits = 0)

# create possible classes

classes <- c(0, 1)

# auxiliary confusion table

tabel <- table(factor(df2$prediction, levels = classes),

factor(df2$gs_ffsstate, levels = classes))

confusion_mat <- confusionMatrix(tabel, positive = "1")

accuracy <- sum(df2$prediction == df2$gs_ffsstate) / nrow(df2)

sensitivity <- sum(df2$gs_ffsstate == 1 & df2$prediction == 1) /

colSums(tabel)[2]

specificity <- sum(df2$gs_ffsstate == 0 & df2$prediction == 0) /

colSums(tabel)[1]

precision <- as.numeric(confusion_mat$byClass[5])

recall <- as.numeric(confusion_mat$byClass[6])

f1score <- as.numeric(confusion_mat$byClass[7])

brier_obj <- brier_nnet(prob_matrix = cbind(1, pred_mat), data = df2)

int_brier <- as.numeric(brier_obj$Int_brier)

brier_set <- brier_obj$Brier

all_weights <- get_weights(trained_model)

nr_weights <- length(unlist(all_weights))

return(list(weights = nr_weights,

node_size = ncol(get_weights(trained_model)[[1]]),

cross_entropy =

result$metrics$loss[result$params$epochs],

accuracy = accuracy,

sensitivity = as.numeric(sensitivity),

specificity = as.numeric(specificity),

Precision = precision, Recall = recall,

F1score = f1score,

Integrated_brier = int_brier,

Brier_scores = brier_set))

}

# model with one hidden layer

fit_keras <- keras_model_sequential()

# Add layers to the model

# here we have logistic activation function for the inputs but also for the outputs

# we create a densely connected ANN to the output

fit_keras %>%

layer_dense(units = 85, activation = 'sigmoid', input_shape = c(129)) %>%

layer_dropout(rate = 0.2) %>%

layer_dense(units = 1, activation = 'sigmoid')

fit_keras %>% compile(

loss = 'binary_crossentropy', # for binary class classification problem

optimizer = optimizer_sgd(lr = 0.2, momentum = 0.9)

#, metrics = c("accuracy")

)

early_stopping <- callback_early_stopping(monitor = 'val_loss', patience = 5)

event_status <- test_ffs_scaled$gs_ffsstate

# create the matrices to be used for keras library

train_x <- as.matrix(training_ffs_long[, c(1:119, 126:135)])

dimnames(train_x) <- NULL # the object must have empty dimnames

train_y <- training_ffs_long$status

test_x <- as.matrix(validation_ffs_long[, c(1:119, 126:135)])

dimnames(test_x) <- NULL # the object must have empty dimnames

test_y <- validation_ffs_long$status

result <- fit_keras %>% fit(

train_x,

train_y,

epochs = 25,

batch_size = 1000,

validation_data = list(test_x, test_y),

class_weight = list("0" = 1, "1" = 1),

callbacks = c(early_stopping)

)

# metrics_model_nn1h <- measures_calculator(trained_model = fit_keras,

# datanew = test_ffs_long,

# real_status = event_status)

#

lifesupport <- factor(levels(test_ffs$lifesupport))

retransplant <- factor(levels(test_ffs$retransplantation))

# create new hypothetic patient

combis <- expand.grid(

retransplantation = retransplant,

lifesupport = lifesupport

)

combis <- combis[c(1, 3, 4), ]

load("df_synth_normal.RData")

df_synth_normal <- df_synth_normal[1:3, ]

df_synth_normal$lifesupport <- combis$lifesupport

df_synth_normal$retransplantation <- combis$retransplantation

times <- seq(0, 10, length.out = 11)

synth_cox_full <- predictSurvProb(object = cox_full,

newdata = df_synth_normal,

times = times)

# synth_rsf_ffs <- predictSurvProb(object = model_rsf,

# newdata = df_synth_normal,

# times = times)

# create hypothetical patient for synthetic scaled

diab <- factor(levels(test_ffs$diab))

retransplant <- factor(levels(test_ffs$retransplantation))

# create new hypothetic patient

combis <- expand.grid(

retransplantation = retransplant,

diab = diab

)

combis <- combis[c(1, 3, 4), ]

load("df_synth_normal.RData")

df_synth_normal <- df_synth_normal[1:3, ]

df_synth_normal$diab <- combis$diab

df_synth_normal$retransplantation <- combis$retransplantation

times <- seq(0, 10, length.out = 11)

synth_cox_full <- predictSurvProb(object = cox_full,

newdata = df_synth_normal,

times = times)

# synth_rsf_ffs <- predictSurvProb(object = model_rsf,

# newdata = df_synth_normal,

# times = times)

# create hypothetical patient for synthetic scaled

diab <- c(0, 1)

retransplant <- c(0, 1)

combis_scaled <- expand.grid(

retransplantation = retransplant,

diabY = diab

)

combis_scaled <- combis_scaled[c(1, 3, 4), ]

load("df_synth_scaled.RData")

df_synth_scaled <- df_synth_scaled[1:3, ] # keep 3 patients from synthetic data

df_synth_scaled$diabY <- combis_scaled$diabY

df_synth_scaled$retransplantationRetransplantation <- combis_scaled$retransplantation

df_synth_scaled$gs_ffs <- c(1, 5, 7)

df_synth_scaled$gs_ffsstate <- c(0, 0, 0)

rownames(df_synth_scaled) <- NULL

data_synth_long <- data_test_creator(df_synth_scaled)

# find hazard

df_new <- data.frame(hazard = predict_proba(fit_keras,

as.matrix(data_synth_long[, c(1:119, 126:135)]),

batch_size = 1000))

df_new$id <- rep(1:3, each = 10) # ids of the patients

groups_new <- split(df_new, f = df_new$id) # create personalized groups per id

group_probs_new <- lapply(groups_new, function(x) {x <- cumprod(1 - x$hazard)})

synth_nn1h <- do.call("rbind", group_probs_new)

synth_nn1h <- cbind(1, synth_nn1h)

load("synth_rsf_ffs.RData")

plot(times, synth_cox_full[1, ],

type = "l",

col = "green", # green

ylim = c(0,1),

lwd = 2,

#cex = 1,

#lty = 1,

xlab = "Time in years since transplantation",

ylab = "Survival probability",

main = "",

xaxt = 'n'

#,main = "Survival Curves for hypothetical patients with NN 2h"

)

# tcl = -0.2

axis(side = 1, at = seq(0, 10, length.out = 11), labels = TRUE)

lines(times, synth_rsf_ffs[1, ], col = "green", lty = 3 , lwd = 2)

lines(times, synth_nn1h[1, ], col = "green", lty = 2, lwd = 2)

lines(times, synth_cox_full[2, ], col = "orange", lty = 1 , lwd = 2) # orange

lines(times, synth_rsf_ffs[2, ], col = "orange", lty = 3, lwd = 2) # orange

lines(times, synth_nn1h[2, ], col = "orange", lty = 2 , lwd = 2) # orange

lines(times, synth_cox_full[3, ], col = "red", lty = 1, lwd = 2) # red

lines(times, synth_rsf_ffs[3, ], col = "red", lty = 3 , lwd = 2) # red

lines(times, synth_nn1h[3, ], col = "red", lty = 2, lwd = 2) # red

legend("bottomleft", legend = c("Cox", "RSF", "NN",

"Ref", "Diabetes Yes", "Re-trans + Diabetes Yes"),

lty = c(1, 3, 2, 1, 1, 1) , col = c("black", "black", "black",

"green", "orange", "red"),

bty = "n", cex = 0.75, border = "white", xjust = 0)

####################################################################

# Comparisons between the models: on 3 patients from the test data

####################################################################

install_packages <- c("pec", "survival", "caret",

"ggplot2", "gridExtra", "hdnom", "keras",

"fastDummies", "randomForestSRC")

for (i in 1:length (install_packages)){

if (!install_packages[i] %in% installed.packages()){

install.packages(install_packages[i])

}

}

library(pec)

library(survival)

library(caret)

library(survcomp)

library(ggplot2)

library(gridExtra)

library(hdnom)

library(keras)

library(fastDummies)

library(randomForestSRC)

library(parallel)

options(rf.cores = 30, mc.cores = 30)

source("the_functions2.R")

load("training_ffs.RData")

#load("training_ffs_extended.RData")

load("training_ffs_scaled.RData")

load("training_ffs_long.RData")

load("test_ffs.RData")

#load("test_ffs_extended.RData")

load("test_ffs_scaled.RData")

load("test_ffs_long.RData")

surv_f <- Surv(gs_ffs, gs_ffsstate) ~ 1

# Cox full

cox_full <- coxph(Surv(gs_ffs, gs_ffsstate) ~.,

data = training_ffs, x = TRUE, y = TRUE)

times <- seq(0, 10, length.out = 11)

test_ffs_try <- test_ffs[c(4, 10000, 20000), ]

synth_cox_full <- predictSurvProb(object = cox_full,

newdata = test_ffs_try[, 1:97],

times = times)

save(synth_cox_full, file = "synth_cox_full.RData")

model_rsf <- rfsrc(Surv(gs_ffs, gs_ffsstate) ~ ., splitrule = "logrank",

nsplit = 5, data = training_ffs, ntree = 300,

split.depth = "all.trees", var.used = "all.trees",

seed = -12345, mtry = 12, nodesize = 50,

sampsize = nrow(training_ffs) / 2,

forest = TRUE, importance = FALSE)

synth_rsf_ffs <- predictSurvProb(object = model_rsf,

newdata = test_ffs_try[, 1:97],

times = times)

save(synth_rsf_ffs, file = "synth_rsf_ffs.RData")

use_session_with_seed(seed = 12345, disable_gpu = TRUE,

disable_parallel_cpu = TRUE,

quiet = FALSE)

# run a neural network with 1 hidden layer

data_test_creator <- function(data){

N <- nrow(data)

# assign survival times to 10 intervals

data$interval <- max(as.numeric(cut(data$gs_ffs,

breaks = 10)))

# the true interval survival

data$survival <- as.numeric(cut(data$gs_ffs,

breaks = 10))

data$id <- 70001:(70000 + N) # define the patient ids abstractly

n.times <- data$interval

data_long <- data[rep(seq_len(N), times = n.times), ]

# create the correct intervals

for(i in unique(data_long$id)) {

n_length <- length(data_long$interval[data_long$id == i])

data_long$interval[data_long$id == i] <- 1:n_length

}

data_long$status <- vector(mode = "numeric",

length = nrow(data_long))

# put indication 1 on status at the intervals on

# which a patient has died

for (i in 1:nrow(data_long)) {

if (data_long$gs_ffsstate[i] == 1 &&

data_long$survival[i] <= data_long$interval[i])

data_long$status[i] <- 1

}

intervals2 <- dummy_cols(as.factor(data_long$interval))

colnames(intervals2) <- gsub(".data", "interval", colnames(intervals2))

data_long <- data.frame(data_long, intervals2[, 2:11])

return(data_long)

}

measures_calculator <- function(trained_model,

datanew, real_status) {

df1 <- data.frame(hazard = predict_proba(trained_model,

as.matrix(datanew[, c(1:119, 126:135)]),

batch_size = 1000))

df1$id <- datanew$id # ids of the patients

df1$survival <- datanew$survival # survival time in years

groups <- split(df1, f = df1$id)

true_surv <- unlist(lapply(groups, function(x) {

surv_obj <- x$survival

true_res <- surv_obj[1]

return(true_res)}

))

group_probs <- lapply(groups, function(x) {

x <- cumprod(1 - x$hazard)})

pred_mat <- do.call("rbind", group_probs)

relative_probs <- vector(mode = "numeric",

length = length(group_probs))

for (i in 1:length(group_probs)){

temp <- group_probs[[i]]

ind <- which(1:10 == true_surv[i])

relative_probs[i] <- temp[ind]

}

N0 <- length(unique(df1$id)) # number of unique persons

# in the data frame create random id numbers

# to label the patients

df2 <- data.frame(relative_probs = relative_probs,

gs_ffs = true_surv,

gs_ffsstate = real_status,

id = (70001):(70000 + N0))

df2$prediction <- 1 - round(df2$relative_probs, digits = 0)

# create possible classes

classes <- c(0, 1)

# auxiliary confusion table

tabel <- table(factor(df2$prediction, levels = classes),

factor(df2$gs_ffsstate, levels = classes))

confusion_mat <- confusionMatrix(tabel, positive = "1")

accuracy <- sum(df2$prediction == df2$gs_ffsstate) / nrow(df2)

sensitivity <- sum(df2$gs_ffsstate == 1 & df2$prediction == 1) /

colSums(tabel)[2]

specificity <- sum(df2$gs_ffsstate == 0 & df2$prediction == 0) /

colSums(tabel)[1]

precision <- as.numeric(confusion_mat$byClass[5])

recall <- as.numeric(confusion_mat$byClass[6])

f1score <- as.numeric(confusion_mat$byClass[7])

brier_obj <- brier_nnet(prob_matrix = cbind(1, pred_mat), data = df2)

int_brier <- as.numeric(brier_obj$Int_brier)

brier_set <- brier_obj$Brier

all_weights <- get_weights(trained_model)

nr_weights <- length(unlist(all_weights))

return(list(weights = nr_weights,

node_size = ncol(get_weights(trained_model)[[1]]),

cross_entropy =

result$metrics$loss[result$params$epochs],

accuracy = accuracy,

sensitivity = as.numeric(sensitivity),

specificity = as.numeric(specificity),

Precision = precision, Recall = recall,

F1score = f1score,

Integrated_brier = int_brier,

Brier_scores = brier_set))

}

# model with one hidden hidden layer

fit_keras <- keras_model_sequential()

# Add layers to the model

# here we have logistic activation function for the inputs but also for the outputs

# we create a densely connected ANN to the output

fit_keras %>%

layer_dense(units = 85, activation = 'sigmoid', input_shape = c(129)) %>%

layer_dropout(rate = 0.2) %>%

layer_dense(units = 1, activation = 'sigmoid')

fit_keras %>% compile(

loss = 'binary_crossentropy', # for binary class classification problem

optimizer = optimizer_sgd(lr = 0.2, momentum = 0.9)

#, metrics = c("accuracy")

)

early_stopping <- callback_early_stopping(monitor = 'val_loss', patience = 5)

event_status <- test_ffs_scaled$gs_ffsstate

# create the matrices to be used for keras library

train_x <- as.matrix(training_ffs_long[, c(1:119, 126:135)])

dimnames(train_x) <- NULL # the object must have empty dimnames

train_y <- training_ffs_long$status

test_x <- as.matrix(validation_ffs_long[, c(1:119, 126:135)])

dimnames(test_x) <- NULL # the object must have empty dimnames

test_y <- validation_ffs_long$status

result <- fit_keras %>% fit(

train_x,

train_y,

epochs = 25,

batch_size = 1000,

validation_data = list(test_x, test_y),

class_weight = list("0" = 1, "1" = 1),

callbacks = c(early_stopping)

)

test_scaled_try <- test_ffs_scaled[c(4, 10000, 20000), ]

data_synth_long <- data_test_creator(test_scaled_try)

# find hazard

df_new <- data.frame(hazard = predict_proba(fit_keras,

as.matrix(data_synth_long[, c(1:119, 126:135)]),

batch_size = 1000))

df_new$id <- rep(1:3, each = 10) # ids of the patients

groups_new <- split(df_new, f = df_new$id) # create personalized groups per id

group_probs_new <- lapply(groups_new, function(x) {x <- cumprod(1 - x$hazard)})

synth_nn1h <- do.call("rbind", group_probs_new)

synth_nn1h <- cbind(1, synth_nn1h)

save(synth_nn1h, file = "synth_nn1h.RData")

# load data

# load("synth_cox_full.RData")

# load("synth_rsf_ffs.RData")

# load("synth_nn1h.RData")

times <- seq(0, 10, length.out = 11)

#png(filename = "comparison_3patients.png")

plot(times, synth_cox_full[1, ],

type = "l",

col = "red",

ylim = c(0,1),

lwd = 2,

#cex = 1,

#lty = 1,

xlab = "Time in years since transplantation",

ylab = "Survival probability",

main = "",

xaxt = 'n'

#,main = "Survival Curves for hypothetical patients with NN 2h"

)

axis(side = 1, at = seq(0, 10, length.out = 11), labels = TRUE)

lines(times, synth_rsf_ffs[1, ], col = "red", lty = 3 , lwd = 2)

lines(times, synth_nn1h[1, ], col = "red", lty = 2 , lwd = 2)

lines(times, synth_cox_full[2, ], col = "green", lty = 1 , lwd = 2)

lines(times, synth_rsf_ffs[2, ], col = "green", lty = 3, lwd = 2)

lines(times, synth_nn1h[2, ], col = "green", lty = 2 , lwd = 2)

lines(times, synth_cox_full[3, ], col = "orange", lty = 1, lwd = 2)

lines(times, synth_rsf_ffs[3, ], col = "orange", lty = 3 , lwd = 2)

lines(times, synth_nn1h[3, ], col = "orange", lty = 2, lwd = 2)

legend("bottomleft", legend = c("Cox", "RSF", "NN",

"patient 1",

"patient 2",

"patient 3"),

lty = c(1, 3, 2, 1, 1, 1) , col = c("black", "black", "black",

"green", "orange", "red"),

bty = "n", cex = 0.75, border = "white", xjust = 0)

#dev.off()
